# A labelled-ubiquicidin antimicrobial peptide for immediate *in situ* optical detection of live bacteria in human alveolar lung tissue†
†Electronic supplementary information (ESI) available: Experimental details and Fig. S1–S5. See DOI: 10.1039/c5sc00960j


**DOI:** 10.1039/c5sc00960j

**Published:** 2015-06-29

**Authors:** Ahsan R. Akram, Nicolaos Avlonitis, Annamaria Lilienkampf, Ana M. Perez-Lopez, Neil McDonald, Sunay V. Chankeshwara, Emma Scholefield, Christopher Haslett, Mark Bradley, Kevin Dhaliwal

**Affiliations:** a Pulmonary Optical Molecular Imaging Group , MRC Centre for Inflammation Research , Queen's Medical Research Institute , 47 Little France Crescent , Edinburgh , EH16 4TJ , UK . Email: Kev.Dhaliwal@ed.ac.uk; b EaStCHEM , The University of Edinburgh School of Chemistry , Joseph Black Building, West Mains Road , Edinburgh EH9 3FJ , UK . Email: mark.bradley@ed.ac.uk

## Abstract

The *in situ* immediate detection of the presence of bacteria in the distal human lung is of significant clinical utility. Herein we describe the development and optimization of a bacterial binding fragment (UBI_29–41_) of the antimicrobial peptide, ubiquicidin (UBI), conjugated to an environmentally sensitive fluorophore to enable rapid live bacterial imaging within human lung tissue. UBI_29–41_ was modified for stability in the presence of human lung bronchoalveolar lavage fluid, for affinity to bacterial membranes and functionality in human lung tissue. The optimized cyclic structure yields an optical molecular Smartprobe for bacterial detection in human lung tissue.

## Abstract 2

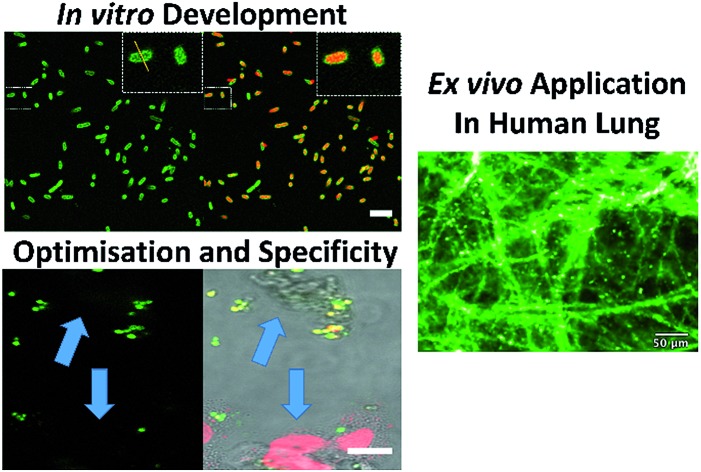
A fluorescently labelled ubiquicidin peptide enables bacterial detection in human lung tissue *in vitro*.

## Introduction

It is a global priority to reduce antibiotic resistance,^1^ and consequently novel methodologies to rapidly diagnose or exclude bacterial infection are urgently required to implement antibiotic stewardship. Current diagnostic approaches rely on detecting the non-specific clinical features of a developing infection alongside optimal sampling of an affected area and subsequent bacterial culture. However, these approaches are slow, prone to contamination and diagnose infection at a late stage. Hence, rapid, sensitive and *in situ* molecular imaging assays for bacterial infections address an unmet need for bacterial diagnostics^2^ and have the potential to lead to more effective antibiotic prescribing.

Pneumonia is an important clinical disease in which to develop and apply bacterial diagnostics. It is the leading infectious cause of mortality in children,^3^ adults admitted to hospital with pneumonia have a mortality of up to 14%,^4^ and in critically ill individuals in the intensive care unit (ICU), those who develop ventilator associated pneumonia (VAP) can have an attributable mortality of over 70% with some etiologies.^5^ During the development of pneumonia, bacteria proliferate within the distal gas exchanging units of the human lung, an anatomical region in humans that is considered to be relatively sterile at basal microbiota levels. This is in contrast to other areas of the human body where bacterial burden from colonisation is high, such as the upper airway and gut, where distinguishing colonisation from pathogenic bacteria is a diagnostic challenge. Indeed current methodologies to diagnose pneumonia rely upon general sampling from upper airways and are prone to contamination and consequent ‘false-positive’ initiation of antibiotic therapy.

Optical molecular imaging (OMI), is a relatively novel diagnostic approach that has utility in preclinical and clinical applications.^6^ Advantages of OMI include: (i) real-time imaging, (ii) high resolution, (iii) absence of radiation-related risks and (iv) low costs. However, whilst the applications of molecular imaging are showing increasing utility in disease areas such as oncology, there are a limited number of functional effective molecular imaging approaches for bacterial detection.^7^

A rational and accepted approach for targeted bacterial molecular imaging has been to label antimicrobial peptides (AMPs), widespread components of the innate immune systems of virtually all multicellular organisms.^8^ AMPs possess a number of factors making them promising tools for bacterial OMI including their amino acid composition, amphipathicity and cationic charge that enable selective insertion and binding to the bacterial membrane.^9^ The most studied AMP for infection imaging is the 59 amino acid cationic AMP, ubiquicidin (UBI) and in particular a UBI fragment containing thirteen amino acid residues (UBI_29–41_) [Thr-Gly-Arg-Ala-Lys-Arg-Arg-Met-Gln-Tyr-Asn-Arg-Arg] which has been utilised in radionucleotide based imaging.^10^ However its use involves unavoidable radiation exposure and is not suitable where portable solutions are required, such as critically ill ventilated patients with suspected pneumonia. More recently a near-infrared labelled version of UBI_29–41_ was applied in a murine model of bacterial infection.^11^ Unfortunately, the synthetic route led to a multitude of products with the dye dominating the distribution of the construct, resulting in a similar distribution for the dye alone compared to the fluorescently labelled UBI_29–41_. In addition there are obvious “Achilles heels” in a labelled UBI_29–41_ peptide with respect to potential degradation *in vivo*, exacerbated even more so in sites of active inflammation by proteolysis and oxidation. Building on these developments and also in recognition of the feasibility of developing an OMI agent (Smartprobe) based upon a UBI scaffold, we embarked upon a lead optimisation approach to yield a Smartprobe suitable for application in diagnosing pneumonia (bacterial infection in the distal lung) when partnered with an optical imaging device. For this Smartprobe to be valuable bio-medically we optimised a series of prerequisites including;

(i) Specificity and selectivity to label clinically relevant bacteria,

(ii) High signal-to-noise contrast,

(iii) Resistance to degradation and stability in the presence of human lung bronchoalveolar lavage fluid (BALF) from patients with acute respiratory distress syndrome (ARDS),

(iv) High affinity and bacterial detection by a clinically approved optical imaging device in human lung alveolar tissue.

We demonstrate the development and optimisation of fluorescently labelled UBI_29–41_, through sequential modification of the fluorophore label, peptide sequence and secondary structure, and demonstrate the ability of the lead compound to detect live bacteria in human lung tissue.

## Results and discussion

We synthesised a series of UBI peptides to assess structure and functional consequences for bacterial labelling (Table 1) and to remove proteolytic susceptibilities.

**Table 1:** Peptide sequences of compounds synthesised with description of modification^*a*^

Compound code	Modification	Peptide sequence	Effect on stability and efficacy
UBI-1	UBI_29–41_ sequence labelled with carboxyfluorescein at the amino-terminus	FAM-Ahx-Thr-Gly-Arg-Ala-Lys-Arg-Arg-Met-Gln-Tyr-Asn-Arg-Arg-NH_2_	Insufficient signal-to-noise
UBI-2	Fluorophore change to NBD	**NBD**-Ahx-Thr-Gly-Arg-Ala-Lys-Arg-Arg-Met-Gln-Tyr-Asn-Arg-Arg-NH_2_	Unstable at 37 °C in PBS
UBI-3	Norleucine substitution of methionine to improve chemical stability	NBD-Ahx-Thr-Gly-Arg-Ala-Lys-Arg-Arg-**Nle**-Gln-Tyr-Asn-Arg-Arg-NH_2_	Unstable in ARDS BALF
UBI-4	*N*-Methylarginine (MeArg) insertion based on MALDI-TOF analysis of cleavage peptides	NBD-Ahx-Thr-Gly-Arg-Ala-Lys-**MeArg-MeArg**-Nle-Gln-Tyr-Asn-**MeArg-MeArg**-NH_2_	Stable in ARDS BALF, poor bacterial affinity
UBI-5	d-Amino acids (d-Val and d-Phe) incorporated at the C-terminus to limit proteolytic degradation	NBD-Ahx-Thr-Gly-Arg-Ala-Lys-Arg-**MeArg**-Nle-Gln-Tyr-Asn-Arg-Arg-**d-****Phe-****d-****Val**-NH_2_	Stable in ARDS BALF, poor bacterial affinity
UBI-6	PEG insertion at the N and C-termini	**MeO-PEG-Lys(NBD)-**Thr-Gly-Arg-Ala-Lys-Arg-Arg-Nle-Gln-Tyr-Asn-Arg-Arg-**PEG-PEG**-NH_2_	Unstable in ARDS BALF, loss of labelling efficacy
UBI-7	PEG introduction at the C-terminus	NBD-Ahx-Thr-Gly-Arg-Ala-Lys-Arg-Arg-Nle-Gln-Tyr-Asn-Arg-Arg-**PEG-PEG**-NH_2_	Unstable in ARDS BALF at 30 minutes, loss of labelling efficacy
UBI-8	The all d-amino acid variant of UBI with the incorporation of d-Nle and a PEG spacer at the amino terminus	NBD**-PEG-****d-****Thr-Gly-****d-****Arg-****d-****Ala-****d-****Lys-****d-****Arg-****d-****Arg-****d-****Nle-****d-****Gln-****d-****Tyr-****d-****Asn-****d-****Arg-****d-****Arg-OH**	Stable in ARDS BALF, loss of labelling efficacy
UBI-9	Retroinverse sequence of UBI (all d-amino acids) and NBD attached to the C-terminal amino acid. Peptide capped at the amino terminus with MeO-PEG	**MeO-PEG-** **d-** **Arg-** **d-** **Arg-** **d-** **Asn-** **d-** **Tyr-** **d-** **Gln-** **d-** **Nle-** **d-** **Arg-** **d-** **Arg-** **d-** **Lys-** **d-** **Ala-** **d-** **Arg-Gly-** **d-** **Thr-Lys(NBD)-OH**	Stable in ARDS BALF, loss of labelling efficacy
UBI-10*	Side-chain to tail cyclisation of UBI with Met to Nle substitution	NBD-Ahx-**cyclo[**Lys-Thr-Gly-Arg-Ala-Lys-Arg-Arg-Nle-Gln-Tyr-Asn-Arg-Arg-Gly]	Improved stability in ARDS BALF, improved bacterial labelling
UBI-11*	As in UBI-10 – a cyclic UBI with Met to Nle substitution and insertion of *N*-MeArg at the major cleavage site	NBD-Ahx-**cyclo[**Lys-Thr-Gly-Arg-Ala-Lys-Arg-Arg-Nle-Gln-Tyr-Asn-Arg-**MeArg**-Gly]	Stable in ARDS BALF, reduction of labelling efficacy

^*a*^Ahx: 6-aminohexanoic acid, PEG: 8-amino-3,6-dioxaoctanoic acid, MeOPEG: 8-methoxy-3,6-dioxaoctanoic acid, FAM: 5(6)-carboxyfluorescein amide, NBD: 7-nitrobenz-2-oxa-1,3-diazole, MeArg: *N*-methyl-arginine, ARDS: acute respiratory distress syndrome. All d-amino acid denoted by prefix D. *A lysine reside was added to the amino terminus of UBI to enable cyclisation.

### 7-Nitrobenz-2-oxa-1,3-diazole (NBD) demonstrates increased signal to noise over carboxyfluorescein (FAM) UBI conjugates

**Fig. 1:**
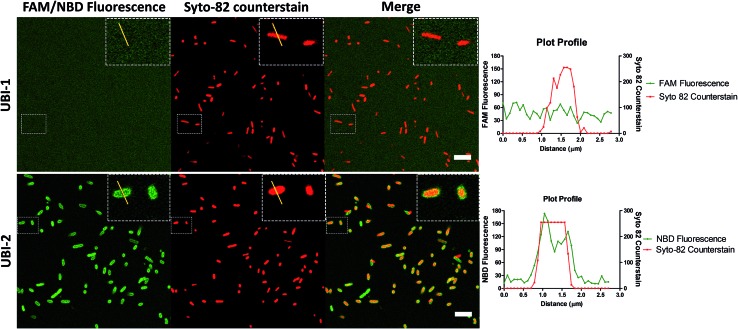
NBD–UBI enables live confocal imaging of bacteria. UBI-2 demonstrates higher signal to noise for bacterial labelling over UBI-1 when imaged *via* confocal microscopy. Upper three panels showing UBI-1 (10 μM) with *P. aeruginosa*; lower panel demonstrates UBI-2 (10 μM) with *P. aeruginosa*. Panel insets show cross section of a single bacterium with a plot profile (right of main panel) across the FAM/NBD image in green and the counterstain in red, demonstrating fluorescence of UBI-2 but not UBI-1 across the bacteria scale bar = 5 μm.

UBI_29–41_ was firstly conjugated to carboxyfluorescein (UBI-1) and used to image bacteria with analysis *via* confocal microscopy. This yielded insufficient signal to noise to distinguish bacteria from background fluorescence (Fig. 1). We therefore took advantage of the environmental reporting properties of the fluorophore 7-nitrobenz-2-oxa-1,3-diazole (NBD)^12^ and hypothesized there would be fluorescence amplification of NBD in the apolar hydrophobic bacterial membrane. UBI-2 demonstrated good signal to noise and was able to detect bacteria (Fig. 1).

### Removing and replacing the methionine residue improves stability

**Fig. 2:**
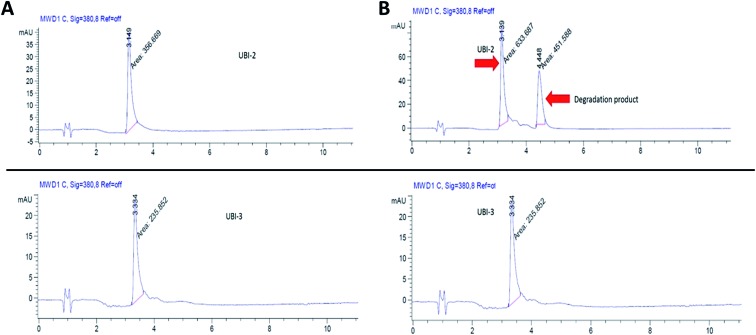
UBI-2 is unstable at 37 °C. HPLC chromatograms (gradient acetonitrile + 0.1% formic acid/water + 0.1% formic acid from 5/95 to 95/5 over 10 min, then isocratic for 4 min; detection at 380 nm). UBI-2 (1 mg mL^–1^ in PBS, upper panels) and UBI-3 (1 mg mL^–1^ in PBS, lower panels) at (A) day 0 and (B) day 3 after incubation at 37 °C.

UBI-2 underwent chemical degradation upon storage in phosphate buffered saline at 37 °C, which was prevented by substitution of the Met to Nle, giving rise to UBI-3 (Fig. 2). This modification is particularly of relevance due to the expected *in vivo* inflammatory oxidative environment within an infected pneumonic lung due to infiltrating host inflammatory cells such as neutrophils,^13^ and therefore all subsequent modifications incorporated Nle in place of Met.

**Fig. 3:**
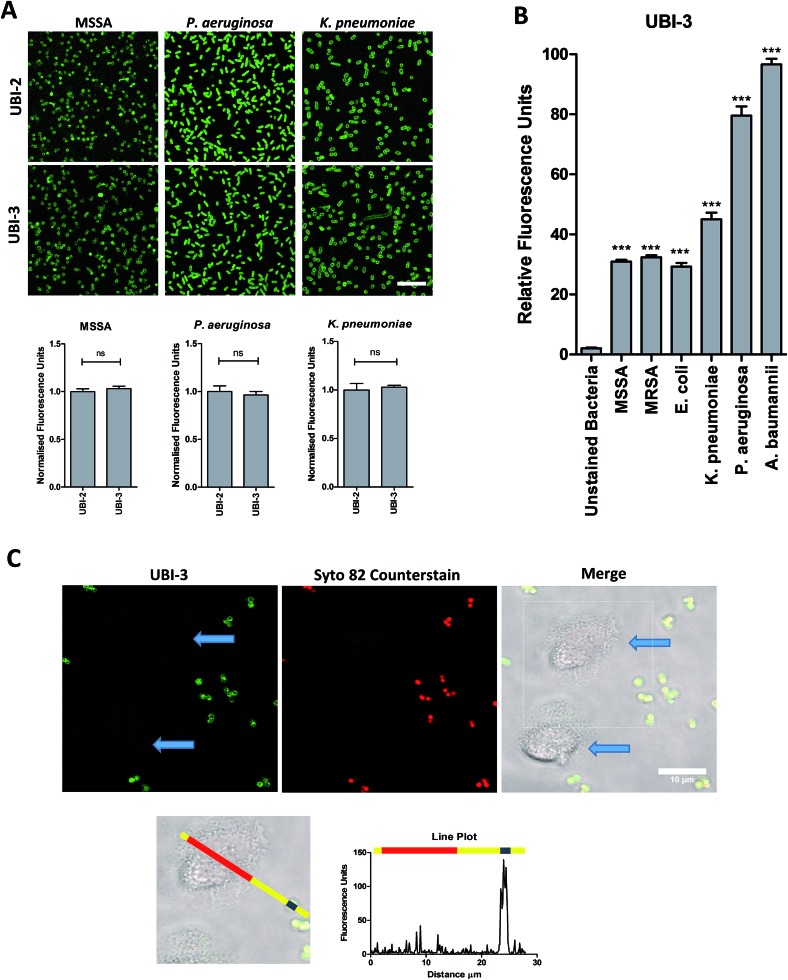
UBI-3 retains specificity and sensitivity for bacterial labelling. (A) Methionine (UBI-2) and norleucine (UBI-3) variants label bacteria with the same fluorescence intensity. Panels show bacteria labelled with either UBI-2 or UBI-3 with quantification of fluorescence (values normalised to UBI-2) (Bars represent mean, error bars represent standard error of mean, *n* ≥ 3, ns = not significant, scale bar = 10 μm). (B) Quantification from confocal images of UBI-3 (10 μM) imaging a panel of bacteria (statistical analyses shown compared to unlabelled bacteria, *n* ≥ 3 for each bacteria, *** = *p* < 0.001). (C) UBI-3; labels bacteria but not isolated human neutrophils (blue arrows). Dotted box enlarged in lower panel and line plot of fluorescence from green channel shown with yellow representing background, red indicating neutrophil area and blue representing single bacteria demonstrating labelling of bacteria but not neutrophils, scale bar = 10 μm. [MRSA: methicillin-resistant *Staphylococcus aureus*, MSSA: methicillin-sensitive *Staphylococcus aureus*].

To assess the bacterial labelling consequences of this single amino acid substitution, UBI-2 and UBI-3 were screened against a panel of Gram positive (methicillin-sensitive *Staphylococcus aureus* (MSSA)) and Gram negative pathogenic bacteria (*Pseudomonas aeruginosa* and *Klebsiella pneumoniae*) demonstrating retained bacterial labelling with specificity over eukaryotic cells (Fig. 3).

### Controlling degradation in human bronchoalveolar lavage fluid (BALF)

**Fig. 4:**
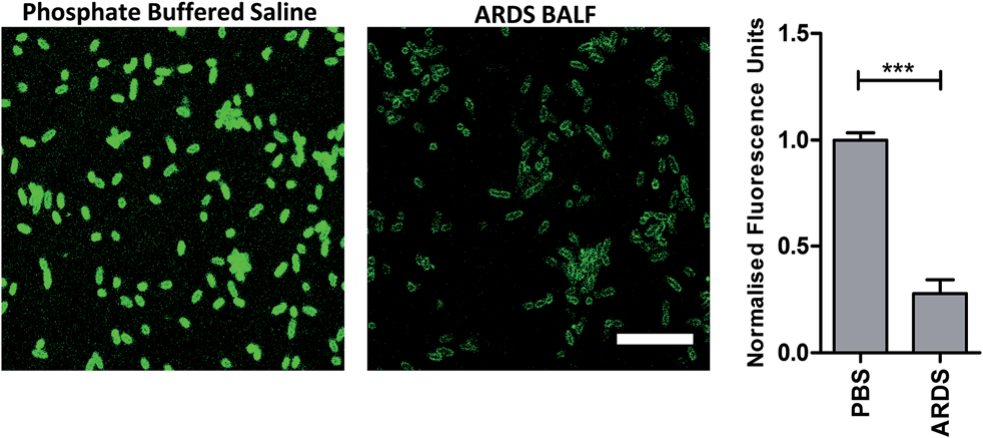
Reduction of fluorescent intensity of *P. aeruginosa* labelled with UBI-3 in the presence of human ARDS BALF. Representative images of *P. aeruginosa* imaged with UBI-3 (10 μM) by confocal microscopy in the presence of phosphate buffered saline (PBS) or ARDS BALF. Quantification of fluorescence intensity demonstrating significantly reduced intensity when compared to PBS (bars represent mean (±SEM), *n* = 3 for PBS and *n* = 5 for ARDS, *** = *p* < 0.001, scale bar = 10 μm).

To determine the ability of UBI-3 to image bacteria in the continued presence of a complex inflammatory environment, bacteria were labelled with UBI-3 in the presence of broncho-alveolar lavage fluid (BALF) retrieved from ventilated ICU patients with Acute Respiratory Distress Syndrome (ARDS). ARDS BALF was utilised as ARDS is a severe life threatening pulmonary syndrome, characterized by a massive proteolytic and oxidative inflammatory environment within the distal alveolar region.^14^ Under these harsh experimental conditions, there was a significant reduction in the fluorescent labelling intensity of bacteria with UBI-3 (Fig. 4). This was due to the rapid (<5 min) degradation of the peptide as demonstrated by Matrix-Assisted Laser Desorption/Ionization Time-of-Flight Mass Spectrometry (MALDI-TOF MS) analysis (ESI Fig. S1†). To design out susceptible amino acid cleavage points, a number of modifications were made (Table 1) and the functional consequences assessed. The chemical approaches pursued to improve resistance to degradation included replacing amino acids at the cleavage positions with non-natural amino acids such as *N*-methyl or d-amino acids,^15^ and cyclisation of the peptide,^16^ which could have the added benefit of enhancing affinity towards bacterial membrane.^17^

### Modification of mass spectrometry identified degradation sites

**Fig. 5:**
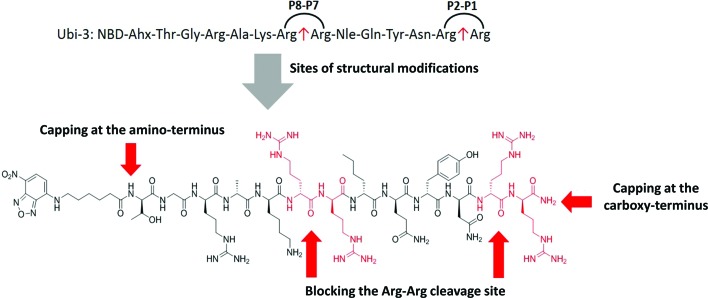
Sites of structural modifications undertaken on UBI-3 based on MALDI-TOF analysis of proteolytic degradation.

**Fig. 6:**
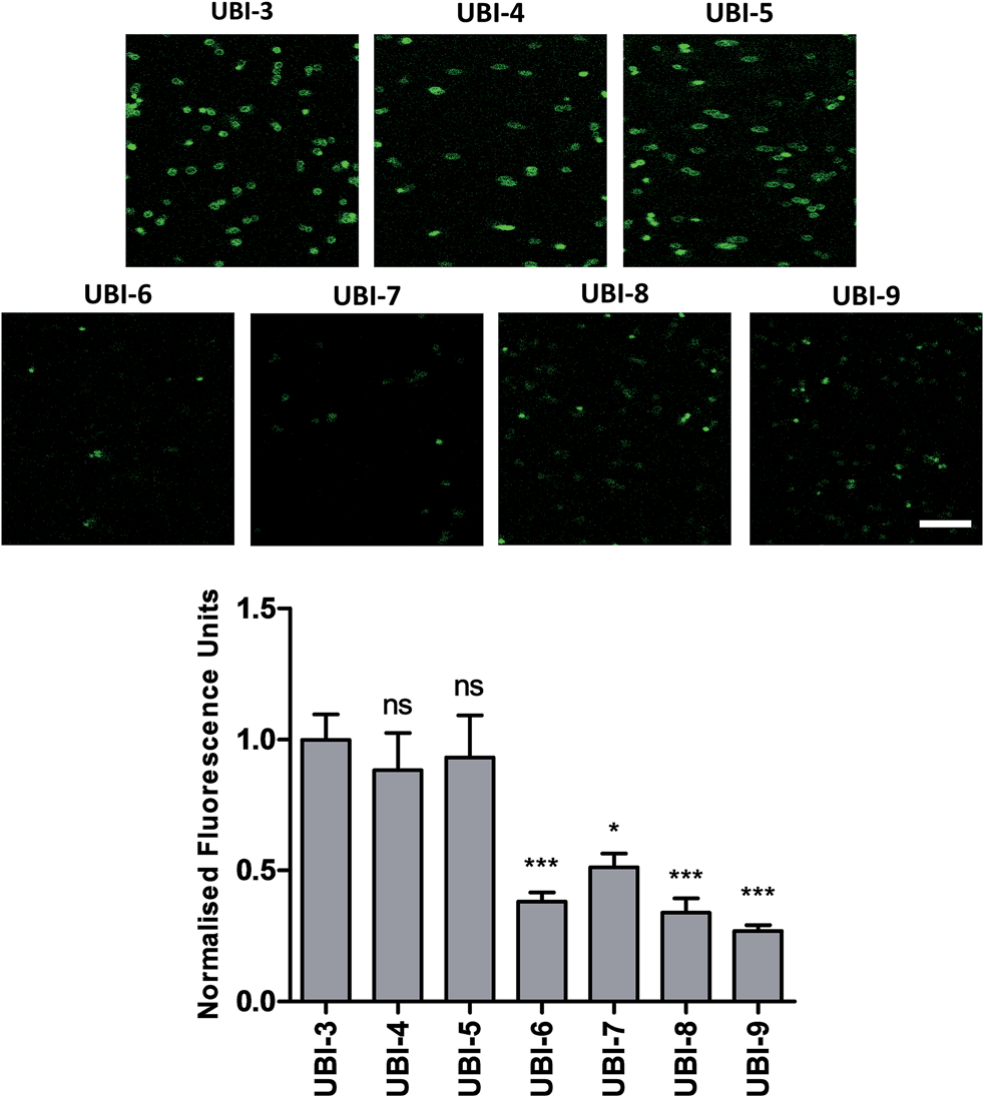
Confocal Images of compounds UBI-3 to 9 with quantified fluorescence. Panels show methicillin-sensitive *Staphylococcus aureus* incubated with UBI-3 to 9 (all 10 μM) with quantification of bacterial fluorescence normalized to UBI-3 (bars represent mean (±SEM), *n* ≥ 3, ns = not significant, *** = *p* < 0.001, * = *p* < 0.05, scale bar = 10 μm).

The Arg–Arg bonds were identified as the prominent position of degradation (Fig. 5) and Compounds UBI-4–9 (Table 1) were synthesised with a variety of substitutions around the degradation sites. The susceptible peptide bonds at P1–P2 and P7–P8 were blocked using *N*-MeArg (UBI-4), and to further mask these positions, a long polyethylene glycol (PEG) chain and/or a d-amino acid were positioned at the carboxylic and/or amino termini (UBI-5–7). *N*-MeArg insertion (UBI-4) or *N*-MeArg with two d-amino acid residues at the C-terminal (UBI-5) improved stability in ARDS BALF (ESI Fig. S2†) and allowed bacterial labelling at the same intensity as UBI-3 (Fig. 6). However, insertion of a PEG chain at both termini (UBI-6) and constructs with entirely d-amino acids (UBI-8 and UBI-9), whilst improving stability, resulted in loss of labelling on bacteria (ESI Fig. S2 and 6†). A single PEG at the C-terminus alone (UBI-7) did not improve stability in ARDS BALF, and resulted in loss of bacterial labelling.

### Cyclisation of the peptide sequence

**Fig. 7:**
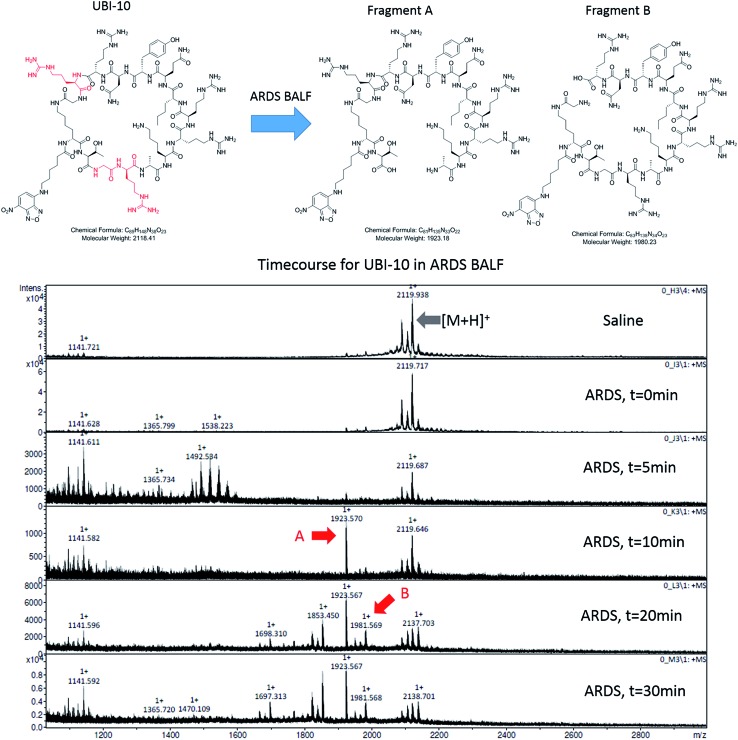
UBI-10 remains stable in ARDS BALF at 5 minutes. (A) Structure of UBI-10 with breakdown products in ARDS BALF. The lower panel shows a MALDI-TOF time course of UBI-10 in ARDS BALF, demonstrating initial stability but the formation of degradation products after 10 minutes.

**Fig. 8:**
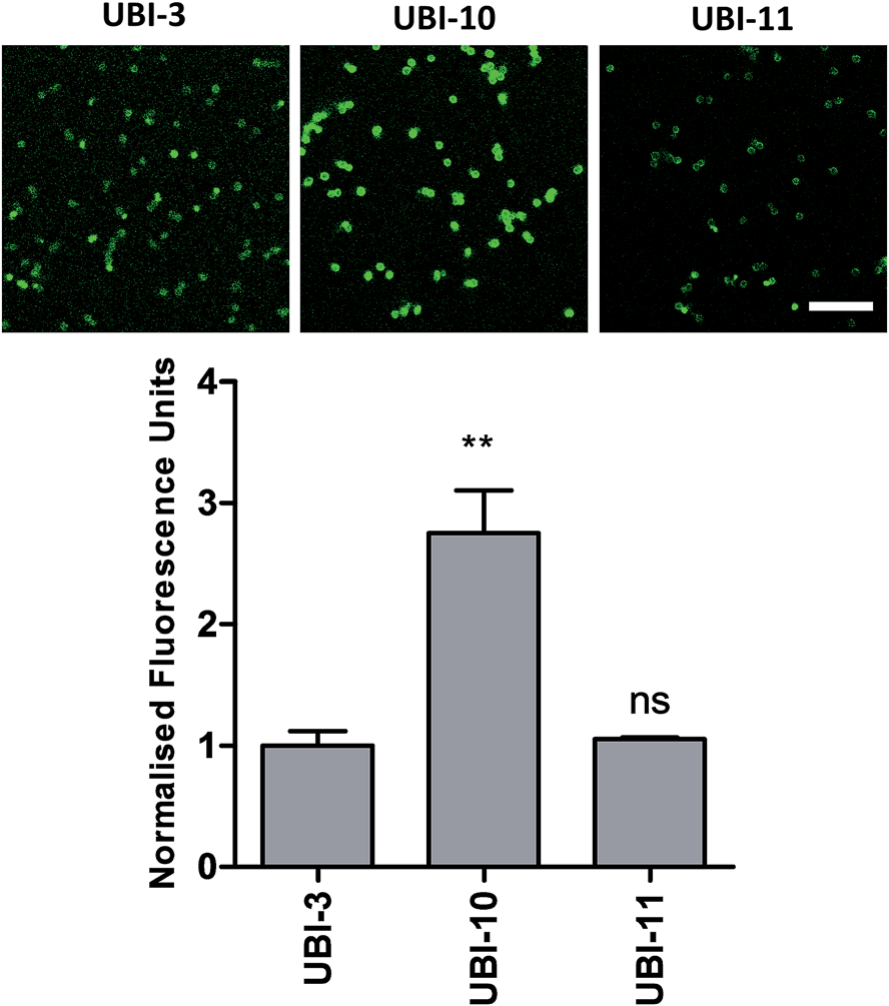
Confocal images and quantification of cyclic variants when compared to UBI-3. Panels show methicillin-sensitive *Staphylococcus aureus* incubated with cyclic UBI compounds (all 10 μM) with quantification of bacterial fluorescence normalized to UBI-3 (bars represent mean (±SEM), *n* ≥ 3, ns = not significant, ** = *p* < 0.01, scale bar = 10 μm).

We hypothesized that cyclisation of the sequence would allow for greater stability and also improve bacterial affinity and therefore cyclic variants of UBI-3 were synthesized (UBI-10 and UBI-11), which were both found to be stable in BALF for 5 minutes (Fig. 7 and S3 ESI†). Compared to the UBI-3 there was a significantly higher signal for equivalent concentrations of the cyclic construct (UBI-10) (Fig. 8). However, these studies also highlighted the degradation of UBI-10 at 10 minutes, guiding the temporal constraints of Smartprobe delivery and optical imaging. UBI-11 demonstrated that a single amino acid change imparted stability in BALF for over 30 minutes and the compound showed comparable bacterial labelling to UBI-3, (ESI Fig. S3†) however the enhanced bacterial labelling seen with UBI-10 was lost (Fig. 8).

### Bacterial affinity evaluation

**Fig. 9:**
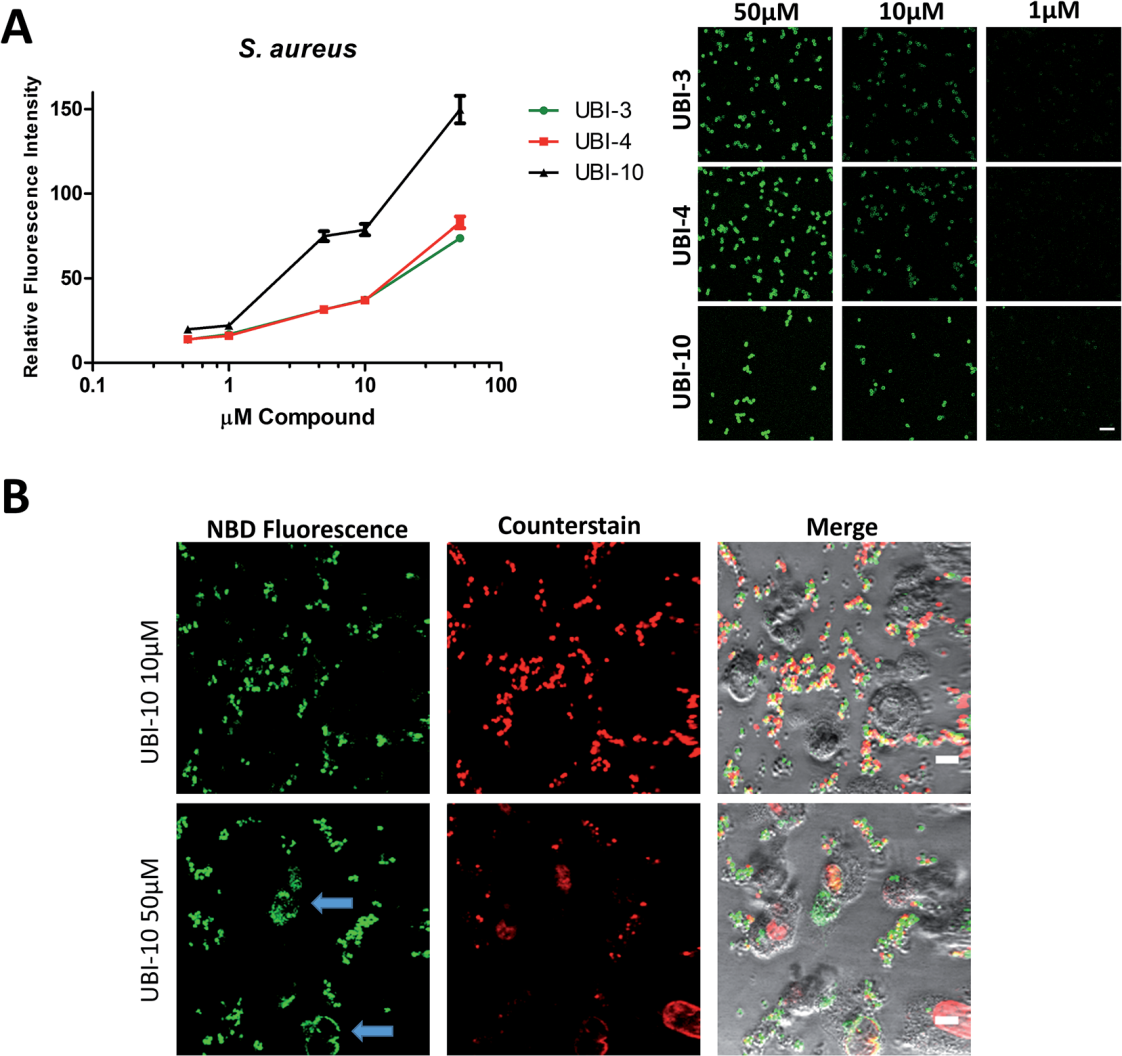
UBI-3, UBI-4 and UBI-10 label bacteria in a concentration dependent manner. (A) UBI-3, UBI-4 and UBI-10 (0.5 μM to 50 μM) co-incubated with methicillin sensitive *S. aureus* and imaged by confocal microscopy with quantification of fluorescence, demonstrating a concentration dependent increase in bacterial fluorescence. UBI-10 retains a higher fluorescence (per molar equivalents) at all concentrations tested. Right panel shows representative images. (B) Representative images of a co-culture of isolated human neutrophils and methicillin sensitive *S. aureus* and UBI-10 at 10 μM (upper panels) and at 50 μM (lower panels) demonstrating no labelling of neutrophils at 10 μM, however at 50 μM, occasional neutrophil labelling was observed (blue arrows).

Three compounds (UBI-3, UBI-5 and UBI-10) were assessed for further evaluation. The rationale for assessing these variants was that UBI-3 and UBI-10 were native UBI sequences in linear or cyclic secondary structures respectively and UBI-5 was stable and demonstrated a similar bacterial labelling to UBI-3. Bacterial labelling was demonstrated in a concentration dependent manner for all three compounds. The relative fluorescence of UBI-10 labelled bacteria was higher than UBI-3 or UBI-5 labelled bacteria (Fig. 9). At 10 μM, UBI-10 retained bacterial specificity over neutrophils. However at 50 μM, some neutrophil labelling was observed (Fig. 9). *In vitro* affinity of bacterial binding was assessed by incorporating a wash step in the live confocal imaging protocol with a panel of bacteria with the ‘wash-off’ representing a surrogate indicator of Smartprobe-bacterial labelling affinity. This demonstrated that following labelling and a wash, there was significantly lower fluorescence for the linear compounds (UBI-3 and UBI-5) than the cyclic compound without modification (UBI-10) (ESI Fig. S4†) against three different bacterial species. UBI-10, therefore, was assessed further in a clinically relevant biological system.

### Bacterial detection by a clinical fibre-based optical imaging in human lung tissue

Fibered confocal fluorescence microscopy (FCFM) with a 488 nm laser system enables *in vivo* cellular and subcellular resolution imaging deep in human lung.^18^ This system allows imaging of the intrinsic autofluorescence of elastin thereby enabling alveolar imaging. Therefore, to assess whether this platform has the resolution to detect fluorescent bacteria, we pre-labeled bacteria (with calcein AM) and then demonstrated that we were able to detect fluorescently labeled bacteria on the background of human lung autofluorescence (ESI Fig. S5†).

**Fig. 10:**
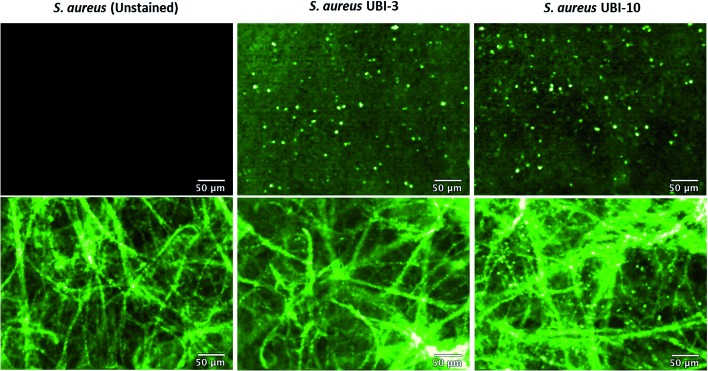
UBI-10 can be used to image bacteria in *ex vivo* human lung tissue by fibered confocal fluorescence microscopy (FCFM). FCFM imaging of bacteria in suspension (upper panels) or when co-incubated with *ex vivo* human lung tissue (lower panels). Left images demonstrate no intrinsic autofluorescence of bacteria, middle panels show UBI-3 can image bacteria in suspension but not in the presence of *ex vivo* human lung and right panels demonstrate UBI-10 can image bacteria by FCFM in the presence of *ex vivo* human lung with a characteristic small round, punctate fluorescence. All compounds at 10 μM, *n* = 3.

Since NBD is also excited at 488 nm, the utility and detection of the Smartprobes with FCFM was next evaluated. Bacteria were labelled with UBI-3 and UBI-10 and imaged in suspension demonstrating the punctate fluorescence pattern of labelled bacteria. These were then delivered onto *ex vivo* human lung tissue fragments. Bacteria labelled with UBI-3 immediately lost their fluorescent signal whereas UBI-10 labelled bacteria, were readily identified above elastin autofluorescence (Fig. 10).

## Summary

We synthesised a library of fluorescently labelled UBI_29–41_ mimetic peptides for rapid, live, bacterial imaging; with the ambition to develop Smartprobes that demonstrate potential clinical utility in conditions of high unmet need; such as the rapid diagnosis of suspected pneumonia in critically ill individuals. We discovered key determinants of peptide stability and bacterial binding affinity as potential obstacles for clinical utility. A number of structural modifications were subsequently engineered to drive improved stability, resistance to degradation and improve function. A cyclic variant of NBD–UBI (UBI-10) proved to be the most functional and specific Smartprobe for bacterial detection and showed proof of concept for detection of bacteria over intrinsic lung elastin autofluorescence in human lung tissue.

These findings provide pivotal insights into future strategies to develop chemical Smartprobes capable to detect bacteria within the distal human lung.

## Supplementary Material

Supplementary information
