# Supplementary material for: A labelled-ubiquicidin antimicrobial peptide for immediate in situ optical detection of live bacteria in human alveolar lung tissue
Source: Chem Sci. 2015 Jun 29;6(12):6971–9. doi: 10.1039/c5sc00960j (PMC5947527; doi:10.1039/c5sc00960j)
Supplement: Supplementary file 1 [file SC-006-C5SC00960J-s001.pdf]

Electronic Supplementary Material (ESI) for Chemical Science.
This journal is © The Royal Society of Chemistry 2015

Supplementary Information

A Labelled-Ubiquicidin Antimicrobial Peptide for Immediate In Situ Optical Detection of Live Bacteria in Human Alveolar Lung Tissue

Ahsan R Akram, Nicolaos Avlonitis, Annamaria Lilienkampf, Ana M Perez-Lopez, Neil McDonald, Sunay V Chankeshwara, Emma Scholefield, Christopher Haslett, Mark Bradley & Kevin Dhaliwal.

Contents:

Figure S1: MALDI-TOF MS spectra for a stability study of UBI-3 in PBS and in ARDS BALF demonstrating breakdown of the compound in ARDS BALF.

Figure S2. MALDI-TOF MS spectra of modified analogues UBI-4 to UBI-9 in Phosphate Buffered Saline and ARDS BALF over a period of 5 and 30min.

Figure S3: MALDI-TOF MS spectra of UBI-11 in Phosphate Buffered Saline vs ARDS BALF over a period of 5 and 30min demonstrating stability.

Figure S4. Quantified fluorescence of confocal microscopy images of the compounds UBI-3, UBI-5 and UBI-10 on three bacteria and fluorescence retention following a PBS wash.

Figure S5: Fibered confocal fluorescence microscopy imaging of human lung and Calcein AM stained Methicillin sensitive *S. aureus.*

Experimental Details including Tables M1-4, Figure M1 and HPLC Chromatograms.

Figure S1: MALDI-TOF MS spectra for a stability study of UBI-3 in PBS and in ARDS BALF demonstrating breakdown of the compound in ARDS BALF.

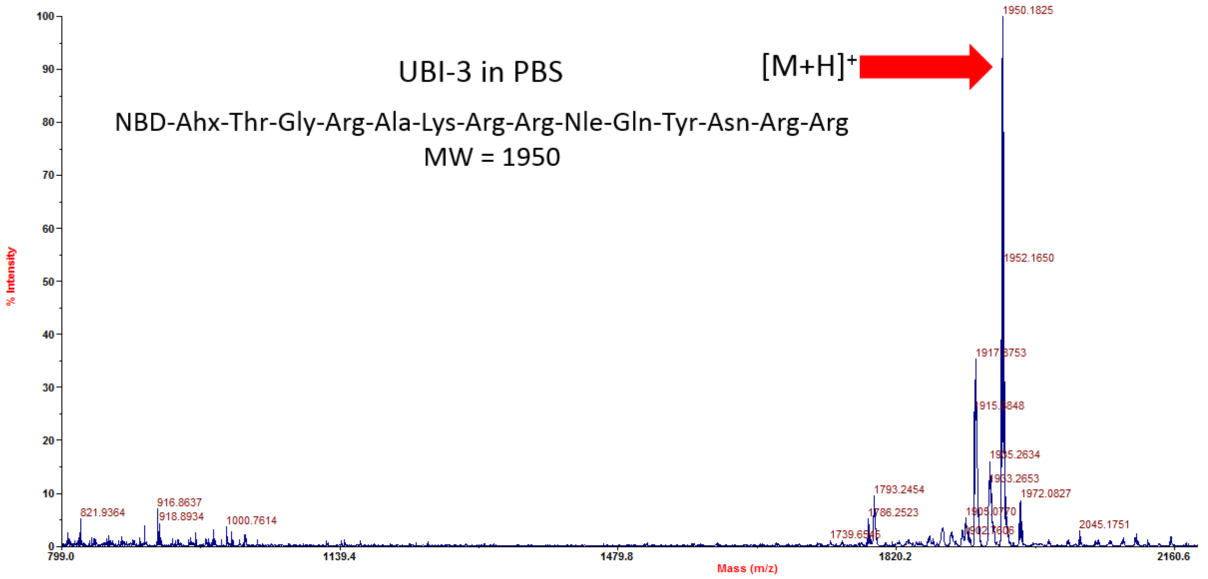

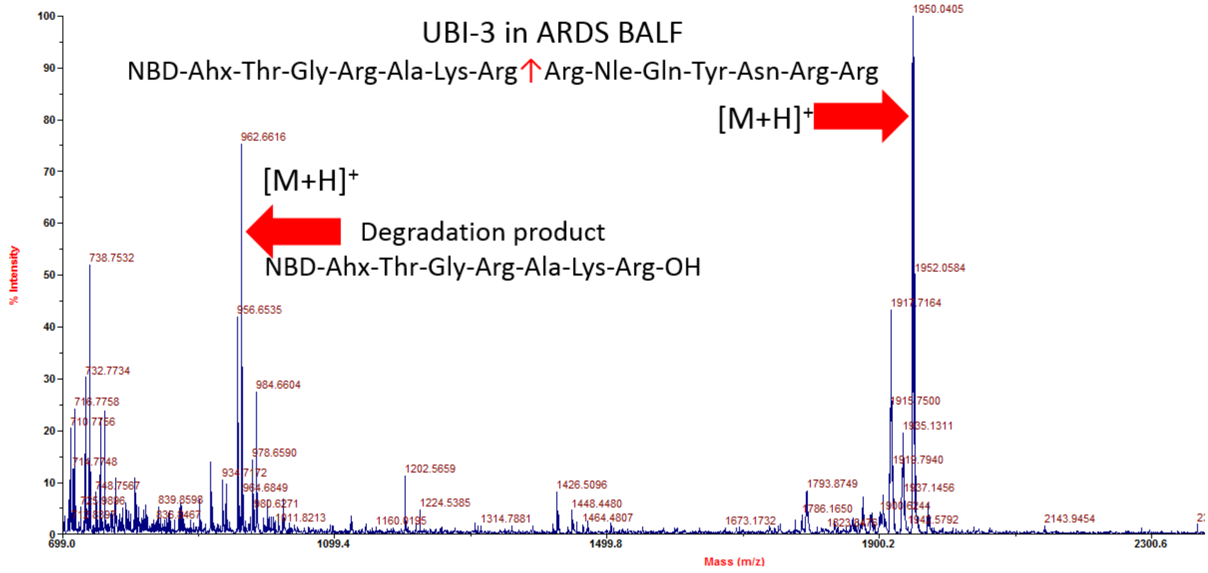

Figure S2. MALDI-TOF MS spectra of modified analogues UBI-4 to UBI-9 in Phosphate Buffered Saline and ARDS BALF over a period of 5 and 30 min.

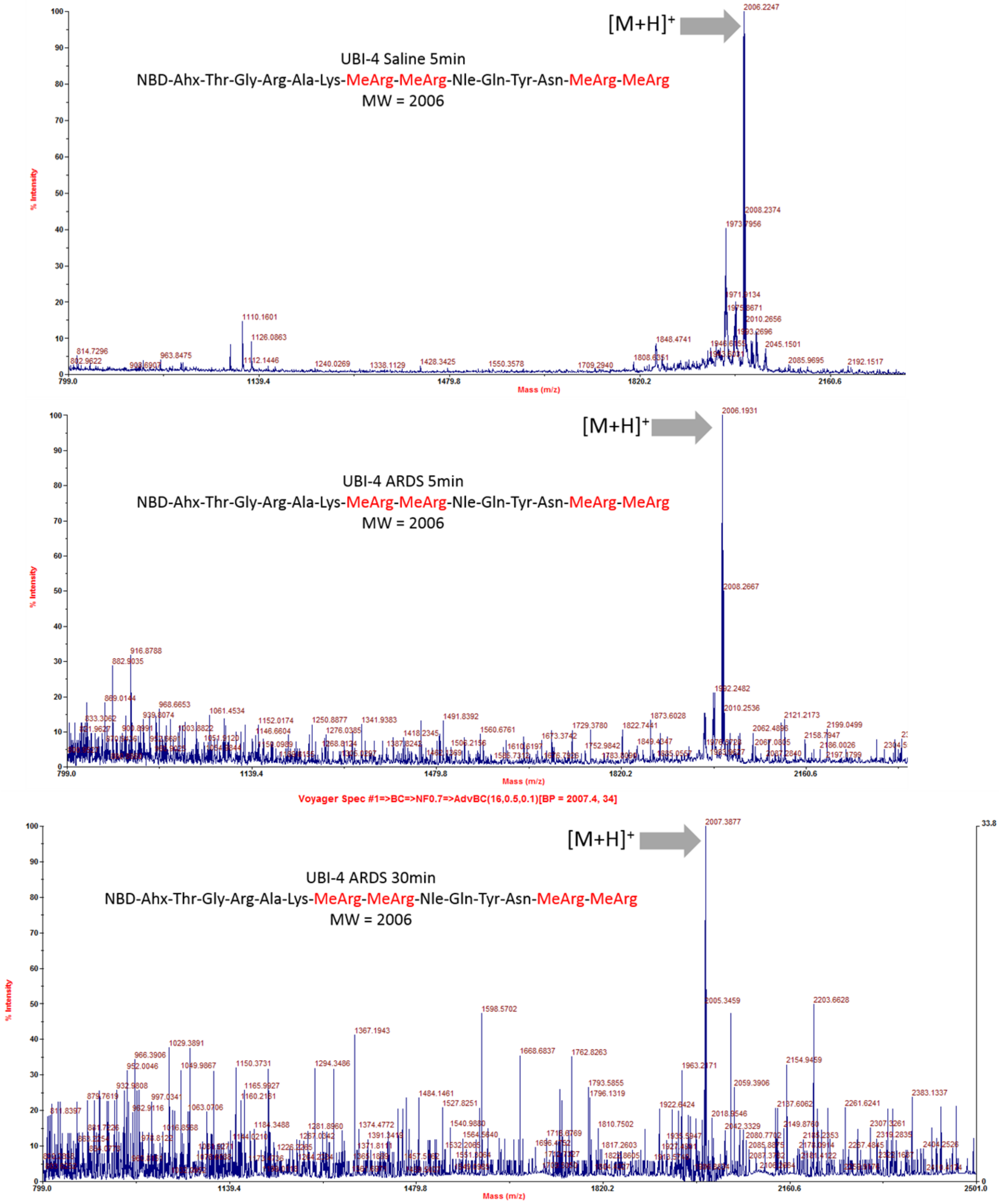

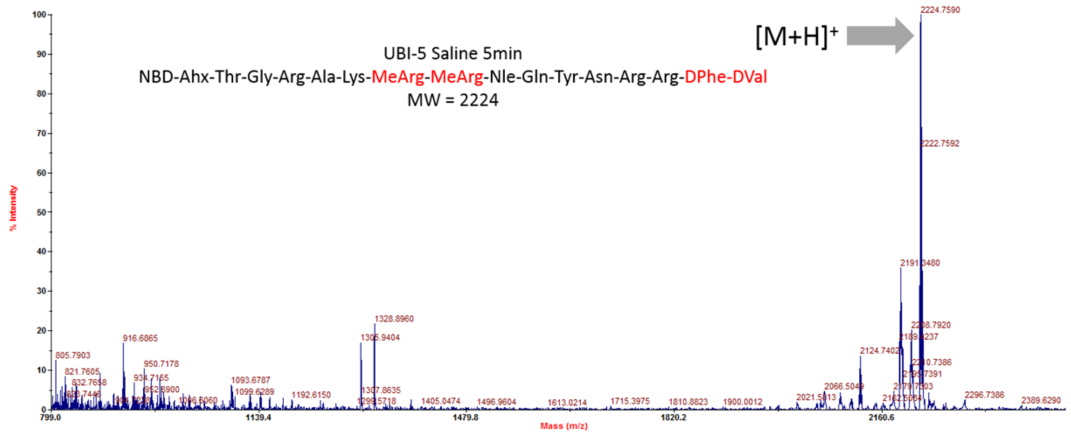
UBI-5 Saline 5min
NBD-Ahx-Thr-Gly-Arg-Ala-Lys-MeArg-MeArg-Nle-Gln-Tyr-Asn-Arg-Arg-DPhe-DVal
MW = 2224
[M+H]+
2224.7590
2222.7592
% Intensity
Mass (m/z)

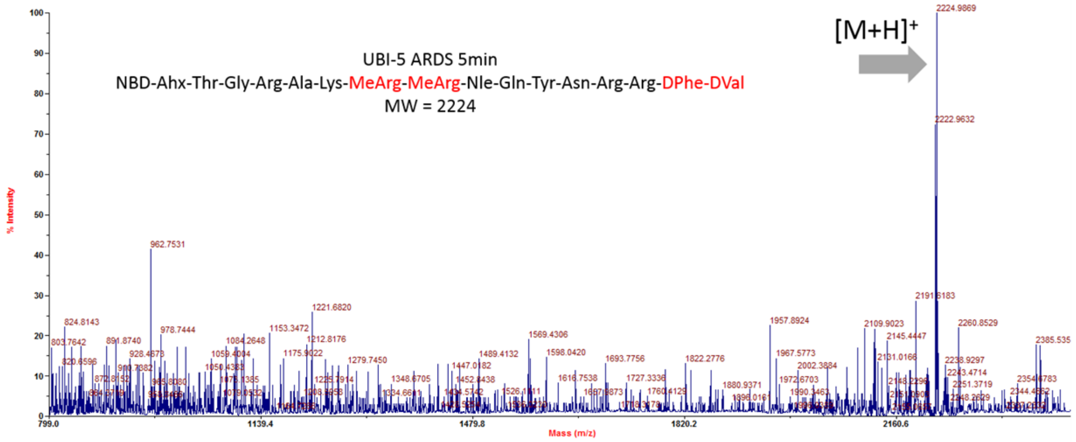
UBI-5 ARDS 5min
NBD-Ahx-Thr-Gly-Arg-Ala-Lys-MeArg-MeArg-Nle-Gln-Tyr-Asn-Arg-Arg-DPhe-DVal
MW = 2224
[M+H]+
2224.9869
2222.9632
962.7531
% Intensity
Mass (m/z)

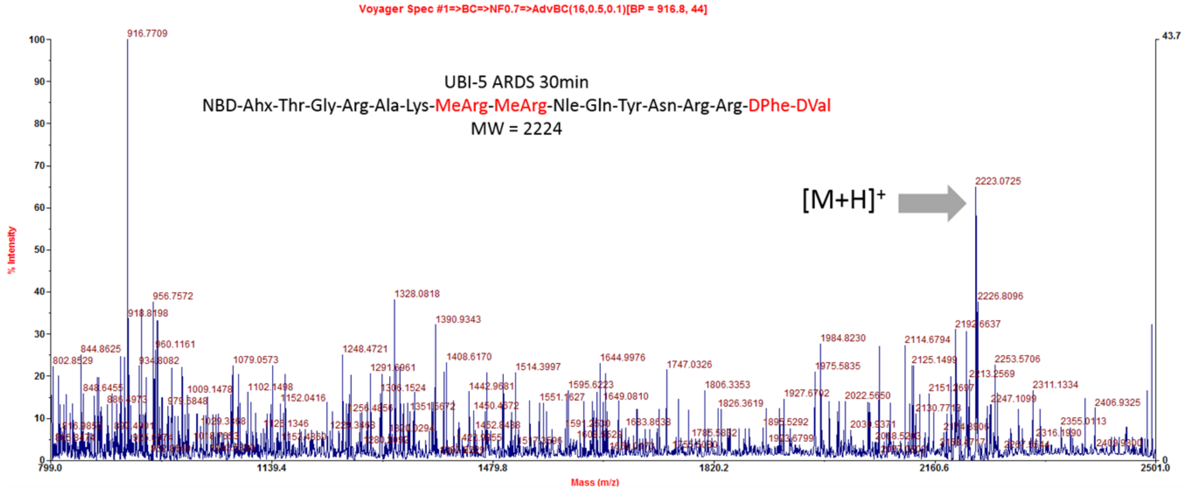
Voyager Spec #1=>BC=>NF0.7=>AdvBC(16,0.5,0.1)[BP = 916.8, 44]
UBI-5 ARDS 30min
NBD-Ahx-Thr-Gly-Arg-Ala-Lys-MeArg-MeArg-Nle-Gln-Tyr-Asn-Arg-Arg-DPhe-DVal
MW = 2224
[M+H]+
2223.0725
916.7709
% Intensity
Mass (m/z)

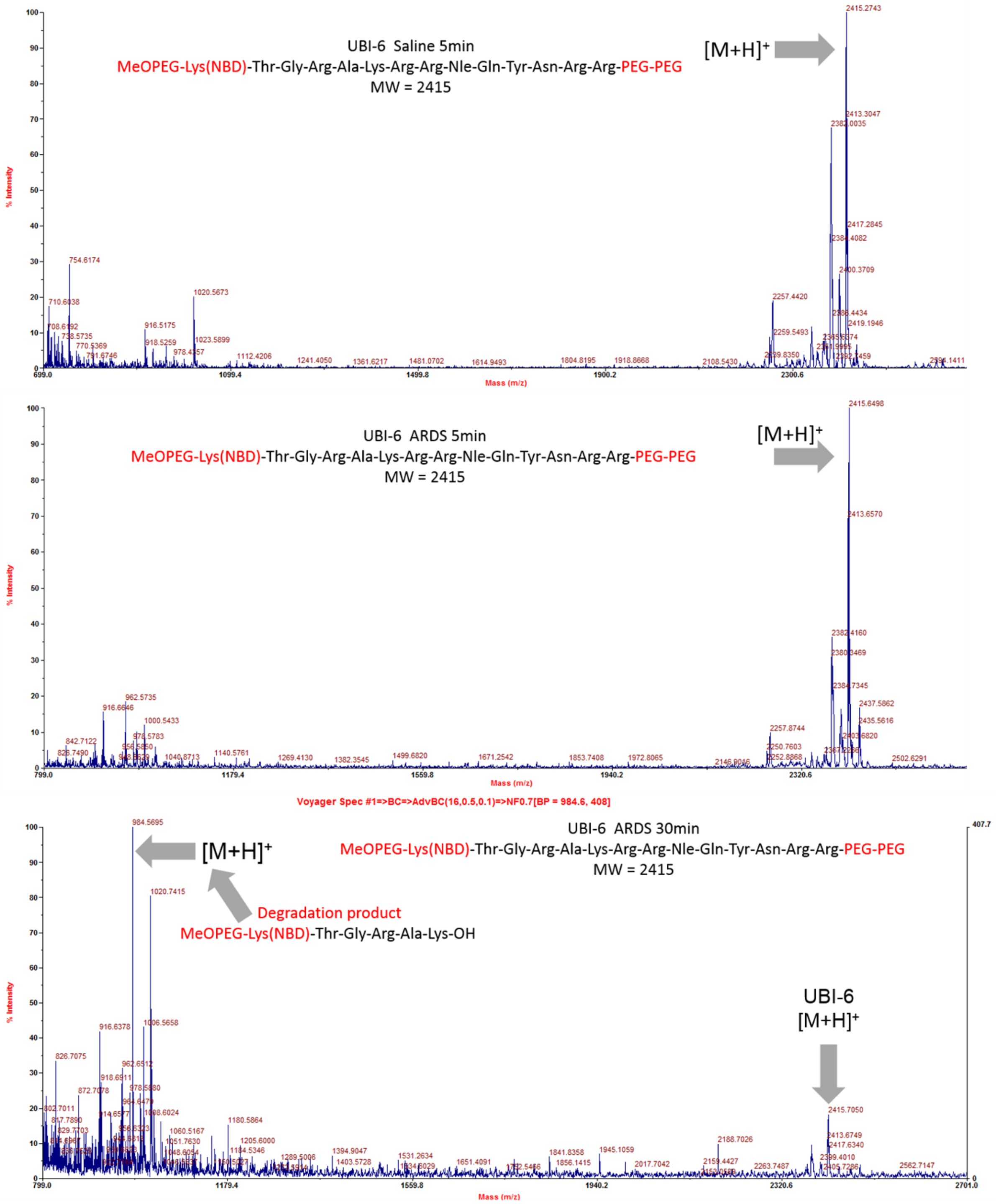

UBI-6 Saline 5min
MeOPEG-Lys(NBD)-Thr-Gly-Arg-Ala-Lys-Arg-Arg-Nle-Gln-Tyr-Asn-Arg-Arg-PEG-PEG
MW = 2415
[M+H]+
% Intensity
Mass (m/z)
UBI-6 ARDS 5min
MeOPEG-Lys(NBD)-Thr-Gly-Arg-Ala-Lys-Arg-Arg-Nle-Gln-Tyr-Asn-Arg-Arg-PEG-PEG
MW = 2415
[M+H]+
% Intensity
Mass (m/z)
Voyager Spec #1=>BC=>AdvBC(16,0.5,0.1)=>NF0.7[BP = 984.6, 408]
UBI-6 ARDS 30min
MeOPEG-Lys(NBD)-Thr-Gly-Arg-Ala-Lys-Arg-Arg-Nle-Gln-Tyr-Asn-Arg-Arg-PEG-PEG
MW = 2415
[M+H]+
Degradation product
MeOPEG-Lys(NBD)-Thr-Gly-Arg-Ala-Lys-OH
UBI-6
[M+H]+
% Intensity
Mass (m/z)

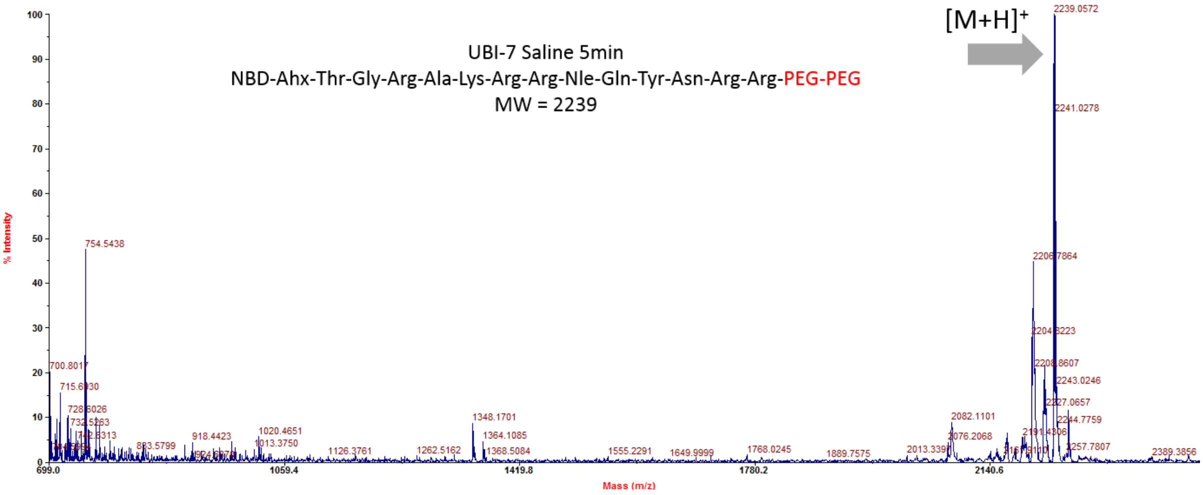
UBI-7 Saline 5min
NBD-Ahx-Thr-Gly-Arg-Ala-Lys-Arg-Arg-Nle-Gln-Tyr-Asn-Arg-Arg-PEG-PEG
MW = 2239
[M+H]+
2239.0572
2241.0278
754.5438
1348.1701
% Intensity
Mass (m/z)
699.0
1059.4
1419.8
1780.2
2140.6

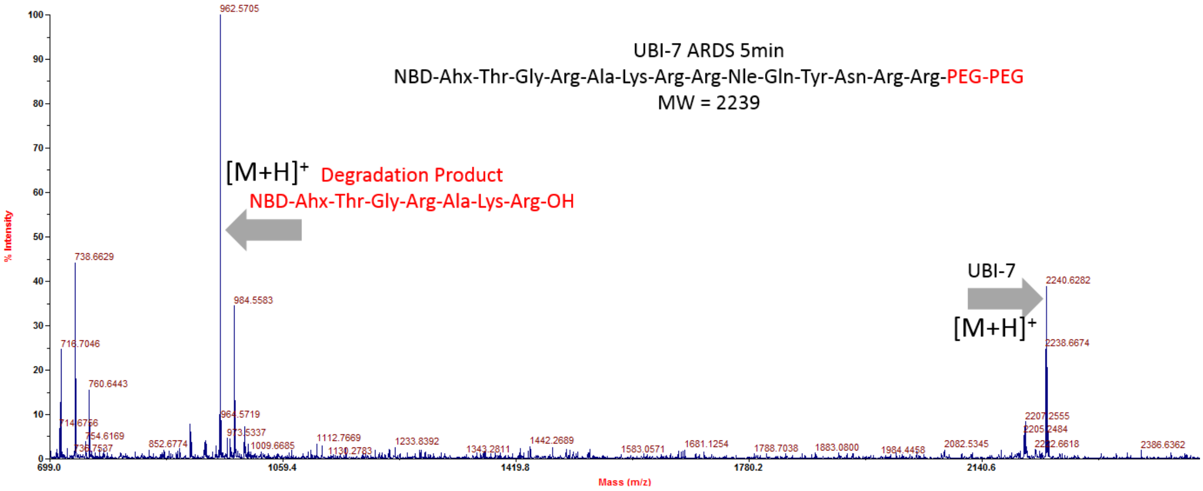
UBI-7 ARDS 5min
NBD-Ahx-Thr-Gly-Arg-Ala-Lys-Arg-Arg-Nle-Gln-Tyr-Asn-Arg-Arg-PEG-PEG
MW = 2239
[M+H]+ Degradation Product
NBD-Ahx-Thr-Gly-Arg-Ala-Lys-Arg-OH
UBI-7
[M+H]+
962.5705
738.6629
984.5583
2240.6282
2238.6674
% Intensity
Mass (m/z)
699.0
1059.4
1419.8
1780.2
2140.6

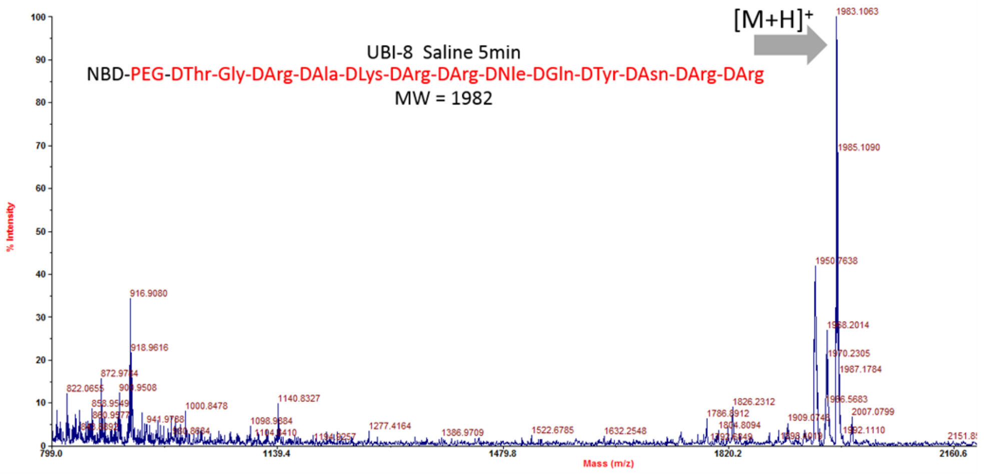

UBI-8 Saline 5min
NBD-PEG-DThr-Gly-DArg-DAla-DLys-DArg-DArg-DNle-DGln-DTyr-DAsn-DArg-DArg
MW = 1982
[M+H]+
1983.1063
1985.1090
916.9080
1140.8327
% Intensity
Mass (m/z)

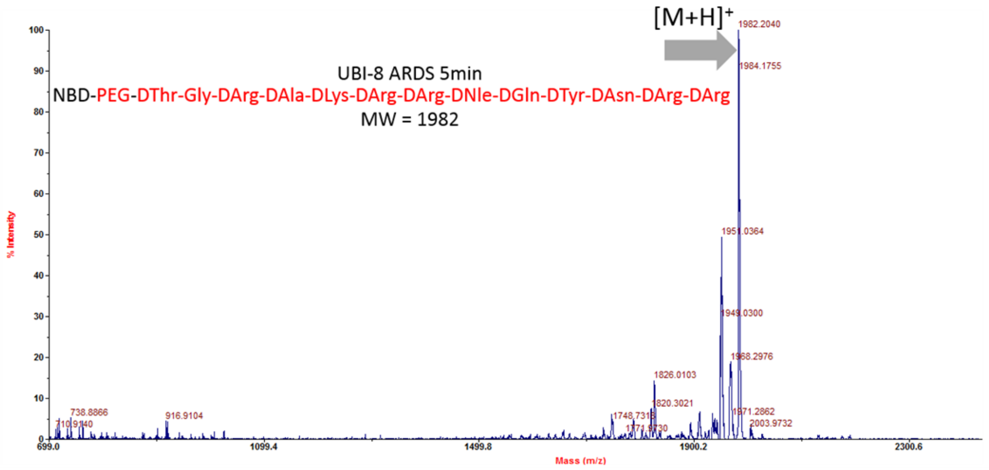

UBI-8 ARDS 5min
NBD-PEG-DThr-Gly-DArg-DAla-DLys-DArg-DArg-DNle-DGln-DTyr-DAsn-DArg-DArg
MW = 1982
[M+H]+
1982.2040
1984.1755
1826.0103
% Intensity
Mass (m/z)

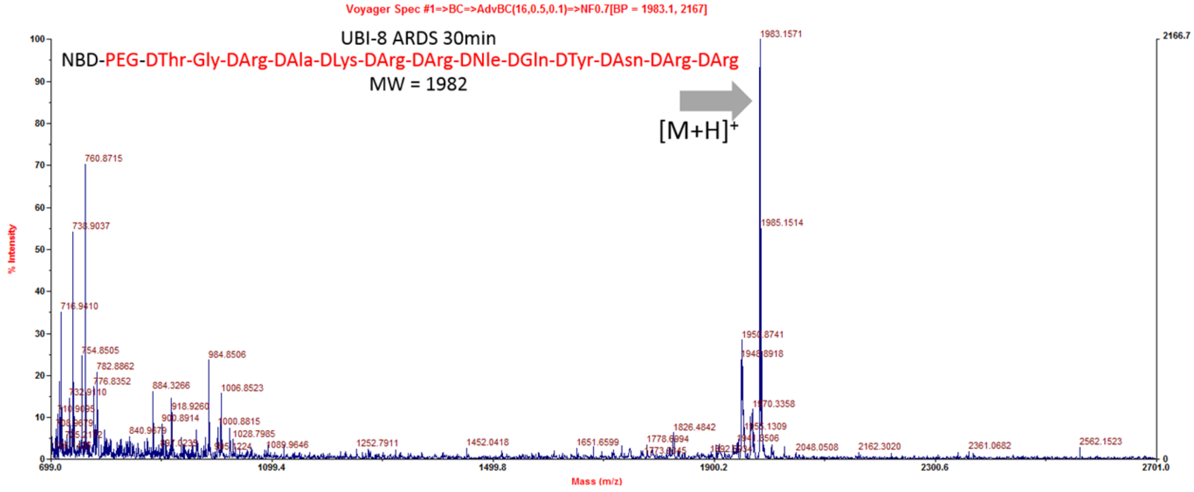

Voyager Spec #1=>BC=>AdvBC(16,0.5,0.1)=>NF0.7[BP = 1983.1, 2167]
UBI-8 ARDS 30min
NBD-PEG-DThr-Gly-DArg-DAla-DLys-DArg-DArg-DNle-DGln-DTyr-DAsn-DArg-DArg
MW = 1982
[M+H]+
1983.1571
1985.1514
760.8715
984.8506
2166.7
% Intensity
Mass (m/z)

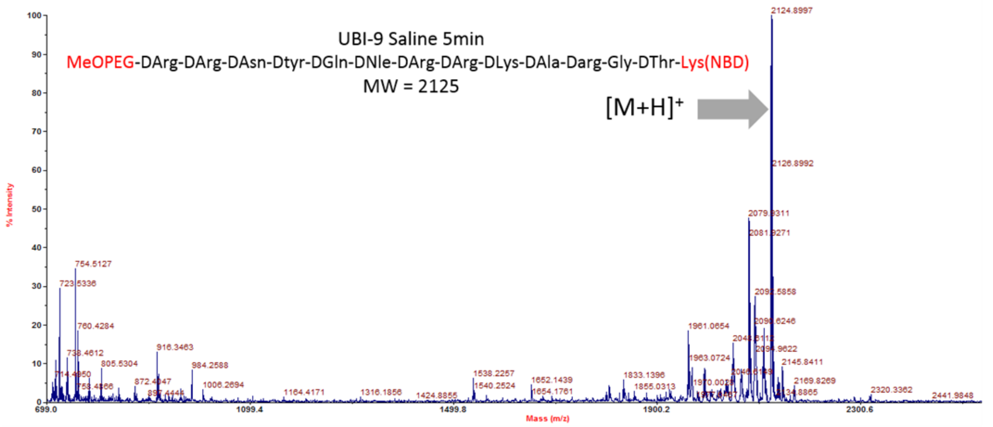
UBI-9 Saline 5min
MeOPEG-DArg-DArg-DAsn-Dtyr-DGln-DNle-DArg-DArg-DLys-DAla-Darg-Gly-DThr-Lys(NBD)
MW = 2125
[M+H]+
2124.8997
2126.8992
2079.8311
754.5127
723.5336
760.4284
916.3463
984.2588
1961.0654
2145.8411
2169.8269
2320.3362
2441.9848
% Intensity
Mass (m/z)

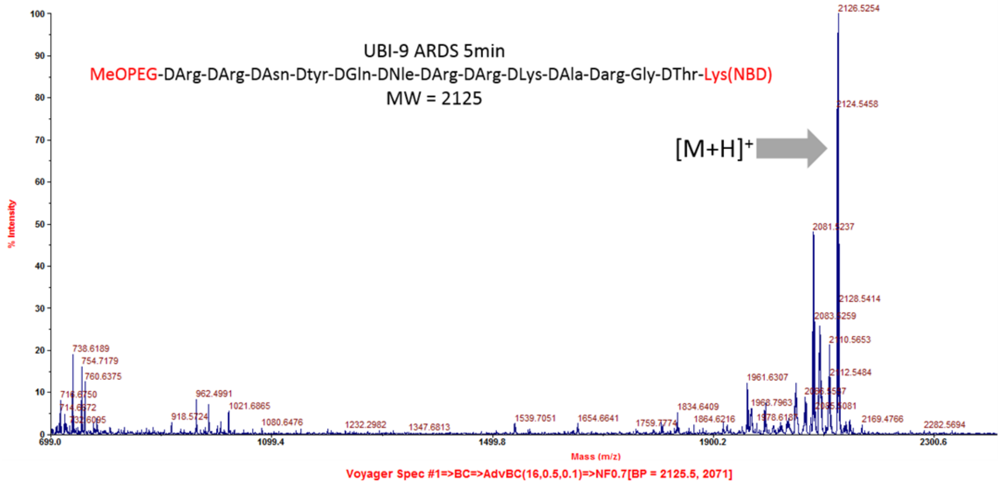
UBI-9 ARDS 5min
MeOPEG-DArg-DArg-DAsn-Dtyr-DGln-DNle-DArg-DArg-DLys-DAla-Darg-Gly-DThr-Lys(NBD)
MW = 2125
[M+H]+
2126.5254
2124.5458
2128.5414
738.6189
754.7179
760.6375
962.4991
1021.6865
1961.6307
2169.4766
2282.5694
% Intensity
Mass (m/z)
Voyager Spec #1=>BC=>AdvBC(16,0.5,0.1)=>NF0.7[BP = 2125.5, 2071]

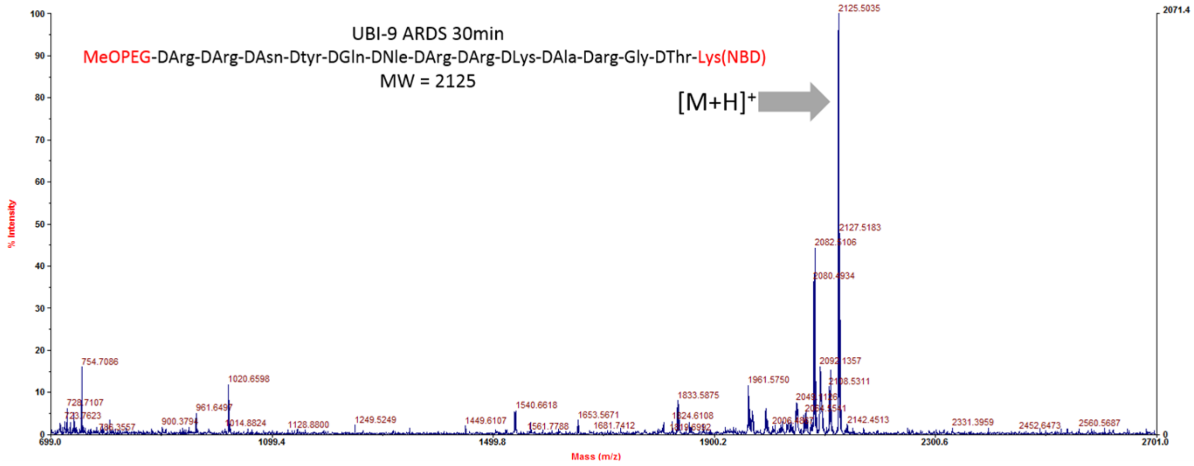
UBI-9 ARDS 30min
MeOPEG-DArg-DArg-DAsn-Dtyr-DGln-DNle-DArg-DArg-DLys-DAla-Darg-Gly-DThr-Lys(NBD)
MW = 2125
[M+H]+
2125.5035
2071.4
2127.5183
754.7086
1020.6598
1540.6618
1833.5875
1961.5750
2142.4513
2331.3959
2452.6473
2560.5687
% Intensity
Mass (m/z)

Figure S3: MALDI-TOF MS spectra of UBI-11 in Phosphate Buffered Saline vs ARDS BALF over a period of 5 and 30min demonstrating stability.

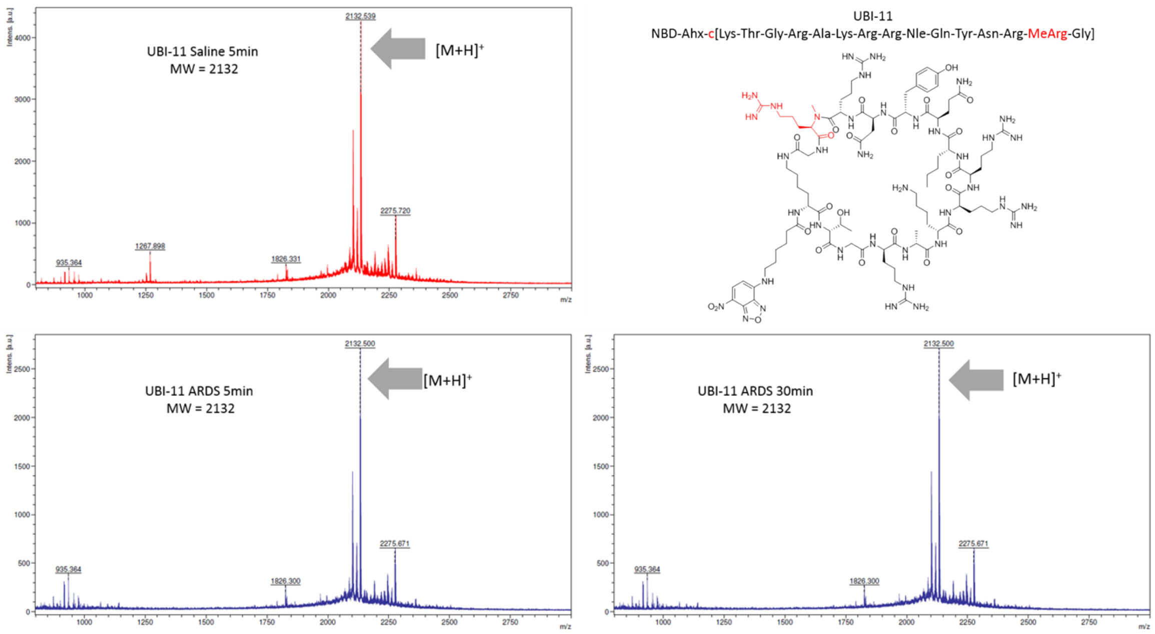

Figure S4. Quantified fluorescence of confocal microscopy images of the compounds UBI-3, UBI-5 and UBI-10 on three bacteria and fluorescence retention following a PBS wash. Quantification of fluorescence intensity of (A) Methicillin sensitive *S. aureus, (*B) *K. pneumoniae and* (C) and *P. aeruginosa* with the three compounds demonstrating only UBI-10 retains a significant fluorescence intensity following a PBS wash. (n=3 for each condition with 10μM compound, error bars represent standard error of mean, ns=not significant, **= p<0.01, ***=p<0.001, statistical analyses when compared to UBI-3).

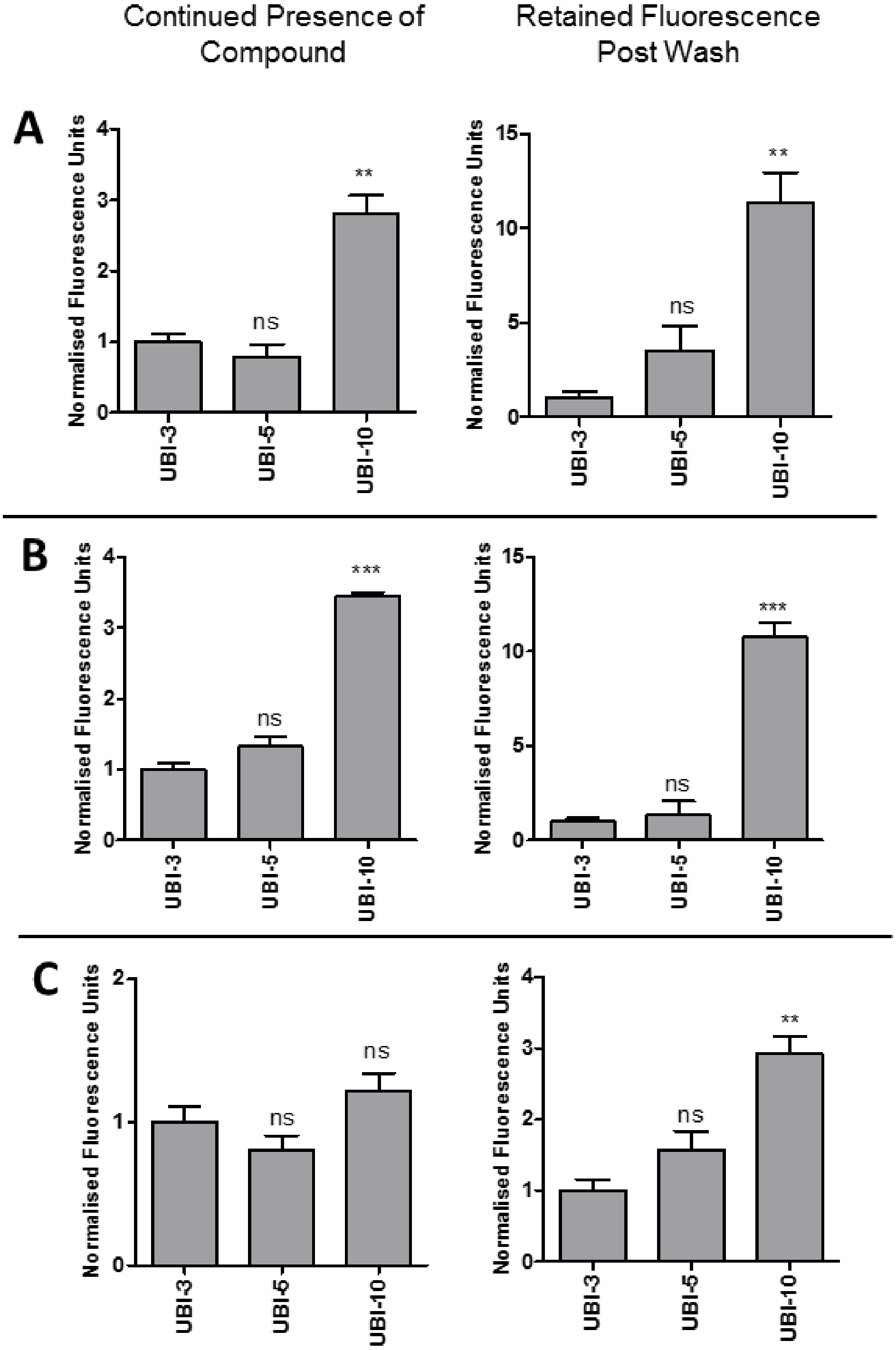

Figure S5: Fibered confocal fluorescence microscopy imaging of human lung and Calcein AM stained Methicillin sensitive *S. aureus.* (A): Demonstrates strong autofluorescent imaging of *ex vivo* human lung tissue; (B) *left*: Demonstrates Calcein AM labelled Methicillin sensitive *S. aureus* in suspension and *right* when added to *ex vivo* human lung tissue. The small round punctate bacterial fluorescence signal is clearly visible (right).

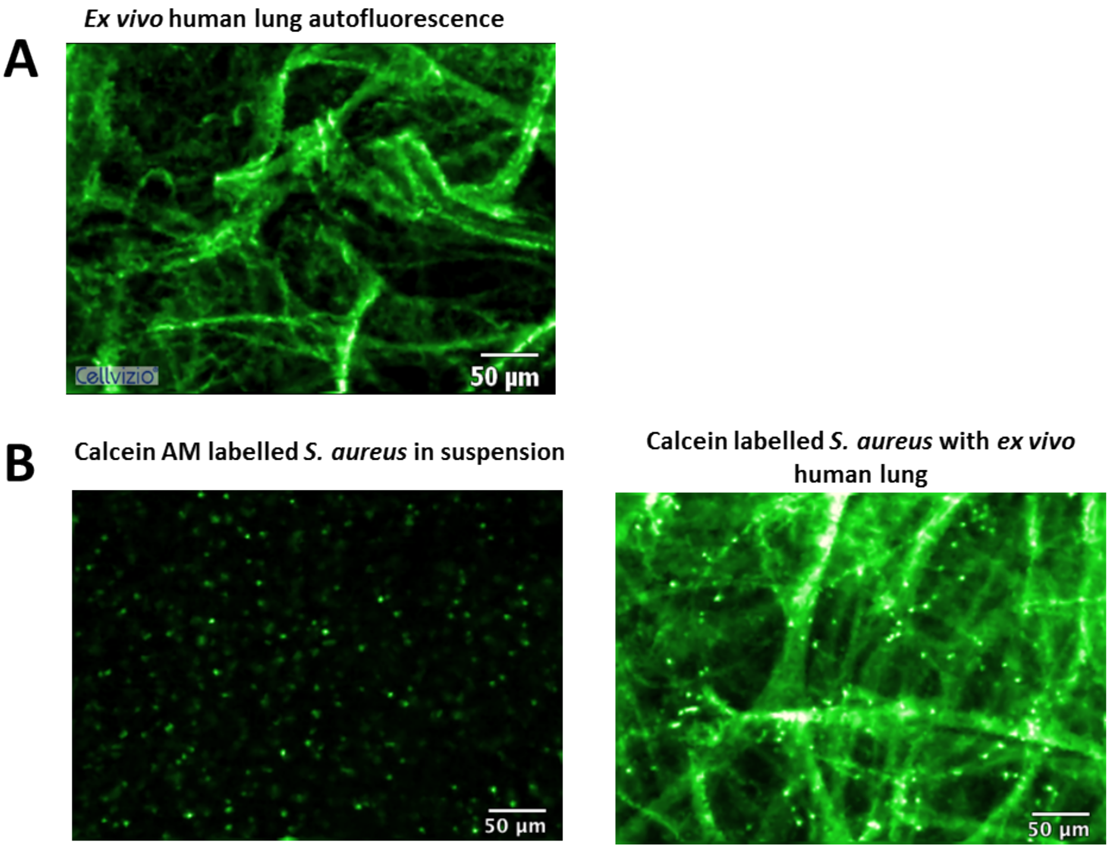

Experimental Details

Chemistry Methods:

All amino acids, aminomethyl Polystyrene Resin (0.745mmol/g, 100~200 mesh, 1% DVB) 2-chlorotrityl chloride resin (1.0mmol/g, 100~200mesh, 1%DVB) and Rink amide linker, HATU were purchased from GL Biochem (Shangai) Ltd and NovaBiochem. H-Gly-2-Chlorotrityl resin (1.1 mmol/g) was purchased from Sigma Aldrich. 4-Chloro-7-nitrobenzofurazan and Oxyma were from Apollo Scientific. 5(6)-carboxyfluorescein was purchased from Merck. *N,N'*-Diisopropylcarbodiimide and diisopropylethylamine were from Sigma-Aldrich, 1-hydroxy-7-azabenzotriazole was TCI Europe. Commercially available reagents were used without further purification.

Analytical reverse-phase high-performance liquid chromatography (RP–HPLC) was performed on an Agilent 1100 system equipped with a Discovery C18 reverse-phase column (5 cm x 4.6 mm, 5 μm) with a flow rate of 1 mL/min and eluting with H_2O/ CH_3CN/HCOOH (95/5/0.05) to H_2O/ CH_3CN/HCOOH (5/95/0.05), over 6 min, holding at 95% CH_3CN for 4 min, with detection at 254 and 495nm and by evaporative light scattering.

Semi-preparative RP–HPLC was performed on an Agilent 1100 system equipped with a Zorbax Eclipse XDB-C18 reverse-phase column (250 x 10 mm, 5 μm) with a flow rate 2.5 mL/min and eluting with 0.1% HCOOH in H_2O (A) and 0.1% HCOOH in CH_3CN (B), with a gradient of 5 to 95% B over 18 min and an initial isocratic period of 4 min.

Electrospray ionization mass spectrometry (ESI–MS) analyses were carried out on an Agilent Technologies LC/MSD Series 1100 quadrupole mass spectrometer (QMS) in an ESI mode.

MALDI TOF MS were run on a Bruker Ultraflextreme MALDI TOF/TOF with a matrix solution of sinapic acid (10 mg/mL) in H_2O/CH_3CN/TFA (50/50/0.1)

Solid Phase Synthesis of UBI-1 to UBI-7

Compound	Sequence
**UBI-1**	FAM-Ahx-Thr-Gly-Arg-Ala-Lys-Arg-Arg-Met-Gln-Tyr-Asn-Arg-Arg-NH_2
**UBI-2**	NBD-Ahx-Thr-Gly-Arg-Ala-Lys-Arg-Arg-Met-Gln-Tyr-Asn-Arg-Arg-NH_2
**UBI-3**	NBD-Ahx-Thr-Gly-Arg-Ala-Lys-Arg-Arg-Nle-Gln-Tyr-Asn-Arg-Arg-NH_2
**UBI-4**	NBD-Ahx-Thr-Gly-Arg-Ala-Lys-MeArg-MeArg-Nle-Gln-Tyr-Asn-NMeArg-MeArg-NH_2
**UBI-5**	NBD-Ahx-Thr-Gly-Arg-Ala-Lys-MeArg-MeArg-Nle-Gln-Tyr-Asn-Arg-Arg-D-Phe-D-Val-NH_2
**UBI-6**	MeO-PEG-Lys(NBD)-Thr-Gly-Arg-Ala-Lys-Arg-Arg-Nle-Gln-Tyr-Asn-Arg-Arg-PEG-PEG-NH_2
**UBI-7**	NBD-Ahx-Thr-Gly-Arg-Ala-Lys-Arg-Arg-Nle-Gln-Tyr-Asn-Arg-Arg-PEG-PEG-NH_2

Table M1: Sequences of UBI-1 to UBI-7; Ahx: 6-aminohexanoic acid, PEG: 8-amino-3,6-dioxaoctanoic acid, MeO-PEG: 8-Methoxy-3,6-dioxaoctanoic acid , FAM: 5(6)-carboxyfluorecein amide, NBD: 7-nitrobenz-2-oxa-1,3-diazole, MeArg: N-Methyl-Arginine, D-Phe: D-Phenylalanine, D-Val: D-Valine

Peptides UBI-1 to UBI-7 were synthesised using standard Fmoc solid-phase synthesis on an aminomethyl polystyrene resin (0.745 mmol/g, 1% DVB, 100-200 mesh) derivatized with 4-[(2,4-dimethoxyphenyl)-(Fmoc-amino)methyl]phenoxyacetic acid (Fmoc-Rink amide linker). The Fmoc-Rink-amide linker (3mmol, 3eq) was dissolved in DMF (0.1M) and ethyl(hydroxyimino)cyanoacetate (Oxyma, 3mmol, 3eq) was added and the mixture was stirred for 10 min. *N,N'*-Diisopropylcarbodiimide (DIC, 3mmol, 3eq) was then added and the resulting mixture was stirred for a further 2 min. The solution was added to aminomethyl polystyrene resin (1mmol, 1eq) and shaken for 3 hours at room temperature. The resulting resin was washed with DMF (x3), DCM (x3) and MeOH (x3). The coupling reaction was monitored by the Kaiser test.[S1]

Fmoc deprotection

To the resin (1mmol) pre-swollen in DCM was added 20% piperidine in DMF (10mL) and the reaction mixture was shaken for 10 min. The solution was drained and the resin was washed with DMF (x3), DCM (x3) and MeOH (x3). This procedure was repeated twice.

Dde deprotection

To the resin (200mg, 0.32mmol), pre-swollen in DCM (5mL), was added 2% hydrazine in DMF (3mL) and the reaction mixture was shaken for 2h. The solution was then drained and the resin was washed with DMF (3×20mL), DCM (3×20mL) and MeOH (3×20mL).

Amino acid coupling

A solution of the appropriate Fmoc-amino acid (3mmol, 3eq) (Fmoc-Ahx-OH, Fmoc-PEG-OH, MeO-PEG-OH, Fmoc-Lys(Dde)-OH, Fmoc-D-Val-OH, Fmoc-D-Phe-OH, Fmoc-Arg(Pbf)-OH, Fmoc-Asn(Trt)-OH, Fmoc-Tyr(tBu)-OH, Fmoc-Gln(Trt)-OH, Fmoc-Met-OH, Fmoc-Nle-OH, Fmoc-Lys(Boc)-OH, Fmoc-Ala-OH, Fmoc-Gly-OH, Fmoc-Thr(tBu)-OH) and ethyl(hydroxyimino)cyanoacetate (Oxyma) (3 mmol, 3eq) in DMF (0.1M) was stirred for 10min. *N,N'*-Diisopropylcarbodiimide (DIC) (3mmol, 3eq) was then added and the resulting solution was stirred for further 2min. The appropriate solution was then added to the resin (1mmol, 1eq), pre-swollen in DCM, and the reaction mixture was shaken for 3 hours at room temperature. The solution was drained and the resin washed DMF (x3), DCM (x3) and MeOH (x3). All coupling reactions were monitored by the Kaiser test.

N-Methyl-Amino acid coupling

The couplings with Fmoc-N-Methyl-Arg(Pbf)-OH and the appropriate Fmoc-Amino acid (Fmoc-Asn(Trt)-OH, Fmoc-Lys(Boc)-OH) onto the peptidyl bound N-Methylated Arginine were performed using microwave irradiation at 65 °C for 10 min (twice) using the following conditions: Fmoc-N-Methyl-Arg(Pbf)-OH (0.38M, 3eq), DIC (2.9eq, 0.35M), 1-hydroxy-7-azabenzotriazole (HAOt) (3.0eq, 0.38M) and N, N'-diisopropylethylamine (DIPEA) (5.8eq, 1.54M). After each coupling step the resin washed with DMF (x3), DCM (x3) and MeOH (x3). All coupling reactions were monitored by the Kaiser test (primary amines) and the chloranil test for secondary amines.[S2]

5(6)-carboxyfluorescein coupling

A solution of 5(6)-carboxyfluorescein (3mmol, 3eq) and Oxyma (3mmol, 3eq) in DMF (0.1M) was stirred for 10 min. DIC (3mmol, 3eq) was then added and the resulting solution was stirred for a further

5min. This solution was added to resin (1mmol, 1eq), pre-swollen in DCM, and the reaction mixture was shaken for 3 h at room temperature. The solution was drained and the resin washed with DMF (×3), DCM (×3) and MeOH (×3). The coupling reaction were monitored by a quantitative ninhydrin test.[S1] Before cleavage, the resin was washed with 20% piperidine to remove any fluorescein phenol esters.[S3]

4-Chloro-7-nitrobenzofurazan (NBD-Cl) coupling

A solution of NBD-Cl (3mmol, 3eq) and DIPEA (3 mmol, 3eq) in DMF (0.1M) was stirred for 1min and then added to the resin (1mmol, 1eq), pre-swollen in DCM, and the reaction mixture was shaken for 3 hours at room temperature. The solution was drained and the resin washed DMF (x3), DCM (x3) and MeOH (x3). The coupling reaction was monitored by the Kaiser test.

Cleavage from the resin

To the resin pre-swollen in DCM was added the cleavage mixture TFA/Phenol/Water/TIS (88/5/5/2) for UBI-1 and UBI-2 or TFA/TIS/DCM (95/2.5/2.5) for UBI-3-7 (1mL/100mg resin) and the mixture was shaken for 3 hours at room temperature. The resin was removed by filtration and the resin was washed with the cleavage mixture once (0.5mL). To the combined filtrates was added dropwise cold diethyl ether to precipitate the crude peptide. This was collected by centrifugation and the diethyl ether was decanted. This solid was washed with diethyl ether three times.

Solid Phase Synthesis of UBI-8 and UBI-9

Compound	Sequence
UBI-8	NBD-PEG-DThr-Gly-DArg-DAla-DLys-DArg-DArg-DNle-DGln-DTyr-DAsn-DArg-DArg-OH
UBI-9	MeO-PEG-DArg-DArg-DAsn-DTyr-DGln-DNle-DArg-DArg-DLys-DAla-DArg-Gly-DThr-Lys(NBD)-OH

Table M2: Sequences of UBI-8 and UBI-9

Peptides UBI-8 and UBI-9 were synthesized by Fmoc-solid phase chemistry using a 2-chlorotrityl chloride linker-resin.

Pre-activation of 2-Chlorotrityl Chloride resin

A polystyrene resin carrying a 2-chlorotrityl linker (CLTR; 50 mg, 0.075 mmol, 1.5 mmol/g) resin was placed into a 5 mL polypropylene syringe fitted with a polyethylene porous frit (20 μm). The resin was swollen with dry DMF (3 × 3 mL) and the DMF removed. A solution of thionyl chloride (20 μL, 0.7 μmol) in DMF (2 mL) was added and the reaction mixture stirred for 1 h. The resin was washed with DMF (3 × 3 mL) and dry DCM (3 × 3 mL) and used immediately or stored at -20 °C for a maximum of 3 days.

First coupling on 2-Chlorotrityl Chloride linker-Resin

The residues (Fmoc-D-Arg(Pbf)-OH or Fmoc-Lys(Dde)-OH) (3 equiv.) were stirred with DIPEA (3 equiv.) in anhydrous DCM (1 mL) for 30 min at room temperature. The resin was washed with DMF (1 mL) and the reactive "chloro" groups were quenched with a solution of DCM:MeOH:DIPEA (2 mL, 80:15:5), followed by washing the resin with DMF (3 × 1 mL).

Cleavage from the resin: Following peptide synthesis the resin was suspended in a cleavage solution of TFA/TIS/Water/Phenol (85:5:5:5), (1 mL) and shaken for 3 h. The resin was removed by filtration and the cleavage solution was evaporated by a flow of air and the peptide re-dissolved in water and lyophilised.

Cyclic peptides (UBI-10 and UBI-11)

Cyclic peptides UBI-10 and UBI-11 were synthesised using a pre-loaded H-Gly-2-Chlorotrityl linker-Resin (1.1 mmol/g) via standard Fmoc solid phase synthesis under microwave irradiation using the conditions given above. At the amino-terminus of the $UBI_{29\text{-}41}$ sequence an extra Lysine residue was added to allow the cyclisation through the lysine side chain and the carboxyl group of the Glycine at the C-terminus. Following the addition of an aminohexanoic acid spacer the peptides were labelled with NBD.

Compound	Sequence
UBI-10	NBD-Ahx-cyclo[Lys-Thr-Gly-Arg-Ala-Lys-Arg-Arg-Nle-Gln-Tyr-Asn-Arg-Arg-Gly]
UBI-11	NBD-Ahx-cyclo[Lys-Thr-Gly-Arg-Ala-Lys-Arg-Arg-Nle-Gln-Tyr-Asn-Arg-MeArg-Gly]

Table M3: Sequences of UBI-10 and UBI-11

Soft cleavage conditions from the resin

To the resin pre-swollen in DCM was added the cleavage mixture TFA/TIS/DCM (1/5/94) (1mL/100mg resin) and the mixture was shaken for 30 min at room temperature. The resin was removed by filtration and the resin was washed with the cleavage mixture once (0.5mL). To the combined filtrates was added dropwise cold diethyl ether to precipitate the crude fully protected peptide. This was collected by centrifugation and the diethyl ether was removed by decantation. The washing procedure with diethyl ether of the full protected peptide was repeated three times. The protected linear peptides were used without further purification.

Cyclisation (General procedure)

The cyclisation was performed by dissolving the protected linear peptide (1mmol, 1eq) and HATU (1mmol, 1eq) in DMF (both at 0.01M). Then, DIPEA (2.5mmol, 2.5eq) was added and the progress of the reaction was followed by RP-HPLC. Upon completion of reaction (~2h), the cyclic peptide was precipitated with water and collected by centrifugation. The washing procedure of the protected cyclic peptide was repeated three times. The yields of the fully protected peptides UBI-10 and UBI-11 were: 86 mg (87%) and 340 mg (40%) respectively. The protected cyclic peptides were treated with the strong TFA conditions (described below) for removal of all the protecting groups

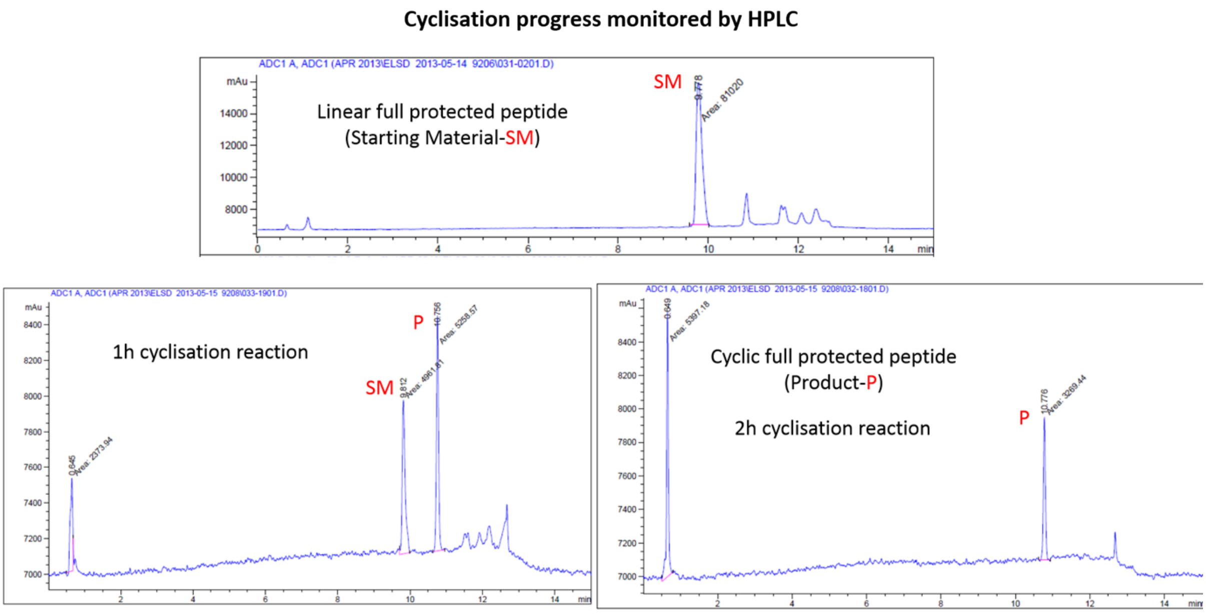

Figure M1: Cyclisation progress monitored by HPLC for the cyclic (protected) UBI-10

Strong TFA conditions for removal of protecting groups

To the cyclic peptide was added the cleavage mixture TFA/TIS/Water/Phenol (85/5/5/5) (1mL/100mg) and the mixture was shaken for 4 hours at room temperature. The deprotected cyclic peptide was concentrated under reduce pressure and added dropwise to cold diethyl ether to precipitate the crude peptide. Semi-preparative HPLC purification was carried out to give the pure cyclic peptides.

Analysis of peptides

Compound	Retention time, min (purity)*	MALDI-TOF, m/z	
		Calculated	Found
UBI-1	2.57 (98%)	2163.42	2164.123 $[M+H]^+$
UBI-2	3.15 (99%)	1968.21	1968.868 $[M+H]^+$
UBI-3	3.33 (99%)	1950.21	1949.982$[M]^+$
UBI-4	2.05 (97%)	2005.13	2006.22 $[M+H]^+$
UBI-5	2.07 (97%)	2223.23	2224.76$[M+H]^+$
UBI-6	2.18 (100%)	2415.71	2415.26 $[M+H]^+$
UBI-7	2.46 (98%)	2239.22	2239.50 $[M]^+$
UBI-8	2.04 (100%)	1984.19	1984.329 $[M+H]^+$
UBI-9	2.10 (100%)	2127.37	2127.670 $[M+H]^+$
UBI-10	2.32 (99%)	2117.16	2118.587 $[M+H]^+$
UBI-11	2.60 (99%)	2160.49	2161.990$[M+H]^+$

Table M4: Peptides – Retention time – m/z (* Detection at 495 nm)

HPLC Chromatograms

UBI-1

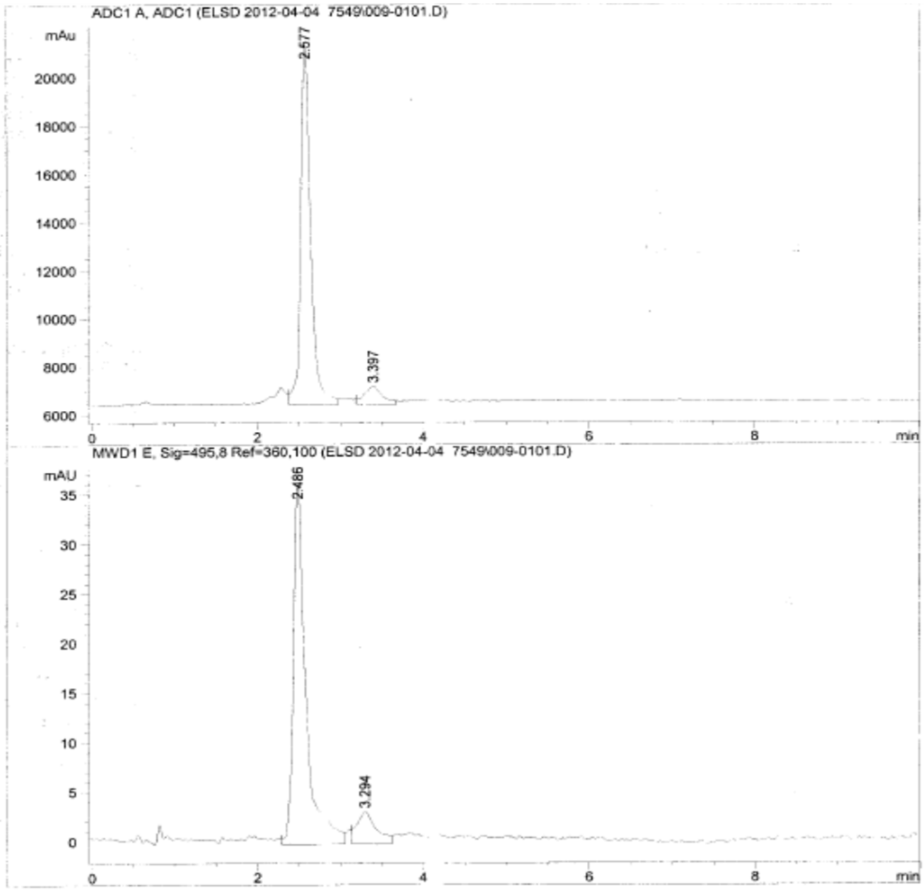

UBI-2

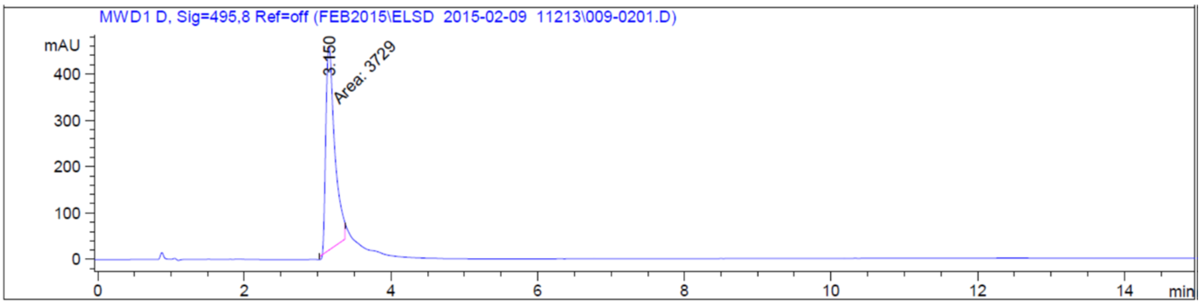

UBI-3

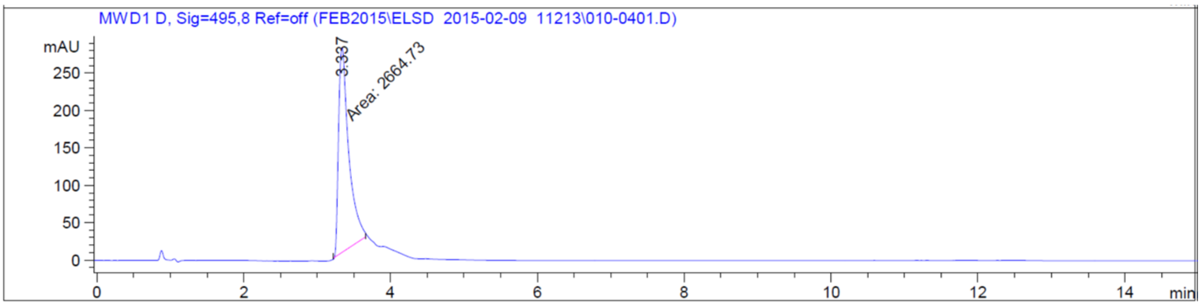

UBI-4

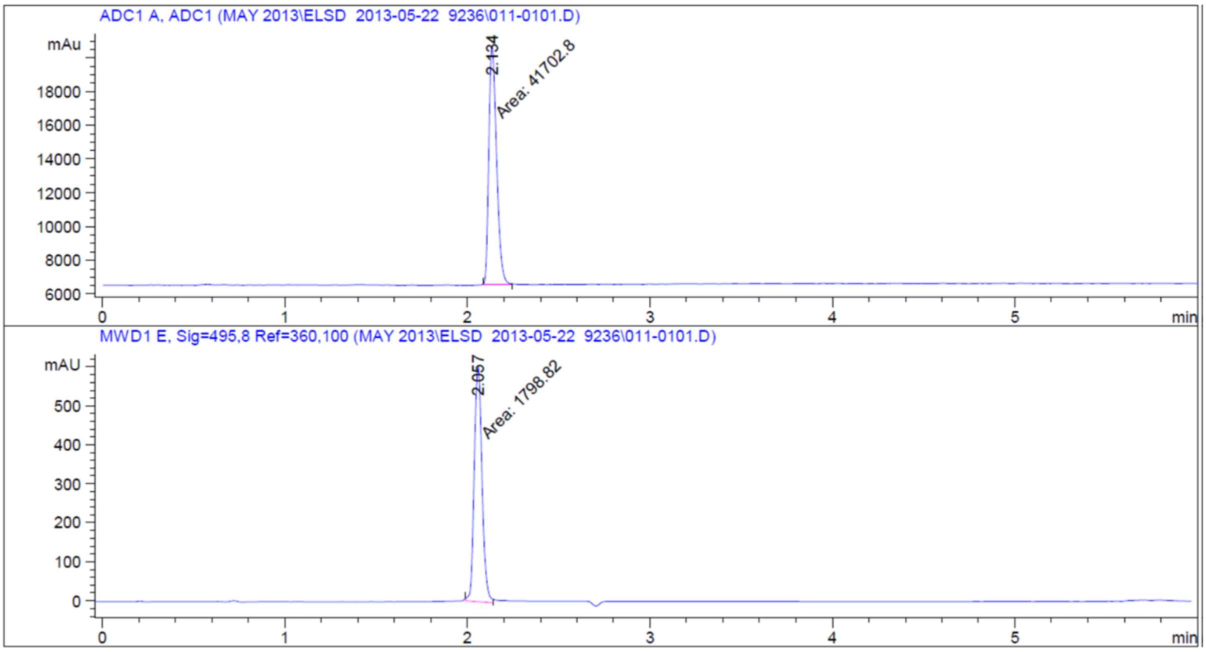
ADC1 A, ADC1 (MAY 2013\ELSD 2013-05-22 9236\011-0101.D)
mAu
2.134
Area: 41702.8
18000
16000
14000
12000
10000
8000
6000
0
1
2
3
4
5
min
MWD1 E, Sig=495,8 Ref=360,100 (MAY 2013\ELSD 2013-05-22 9236\011-0101.D)
mAU
2.057
Area: 1798.82
500
400
300
200
100
0
0
1
2
3
4
5
min

UBI-5

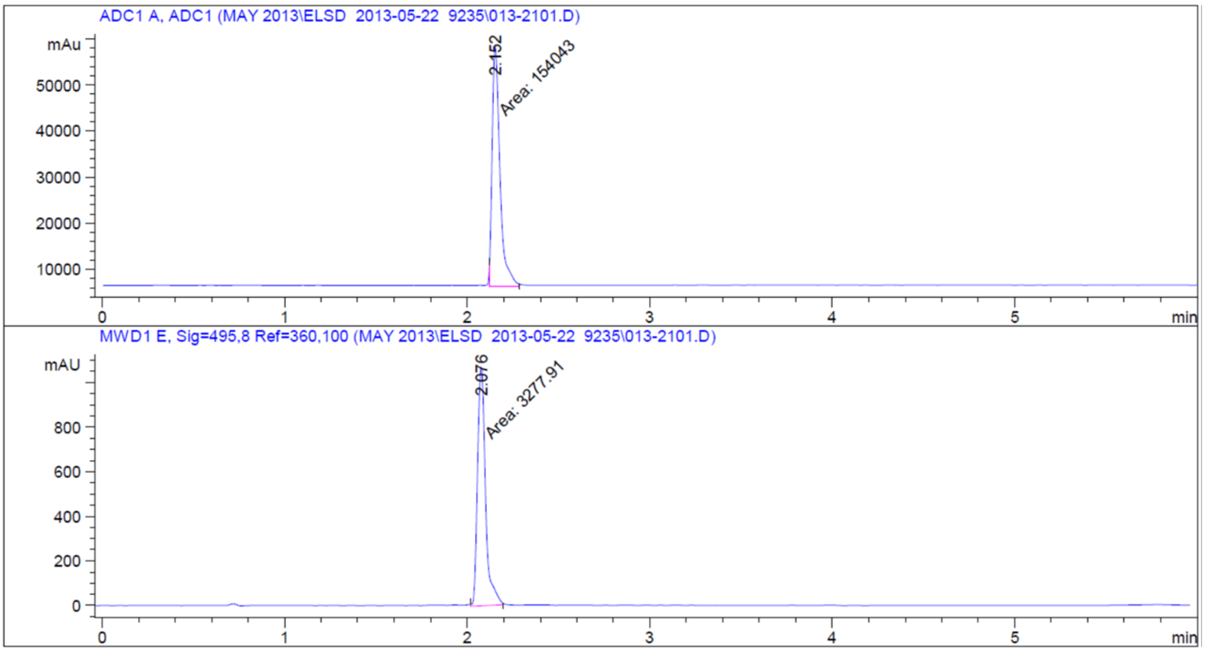
ADC1 A, ADC1 (MAY 2013\ELSD 2013-05-22 9235\013-2101.D)
mAu
2.152
Area: 154043
50000
40000
30000
20000
10000
0
1
2
3
4
5
min
MWD1 E, Sig=495,8 Ref=360,100 (MAY 2013\ELSD 2013-05-22 9235\013-2101.D)
mAU
2.076
Area: 3277.91
800
600
400
200
0
0
1
2
3
4
5
min

UBI-6

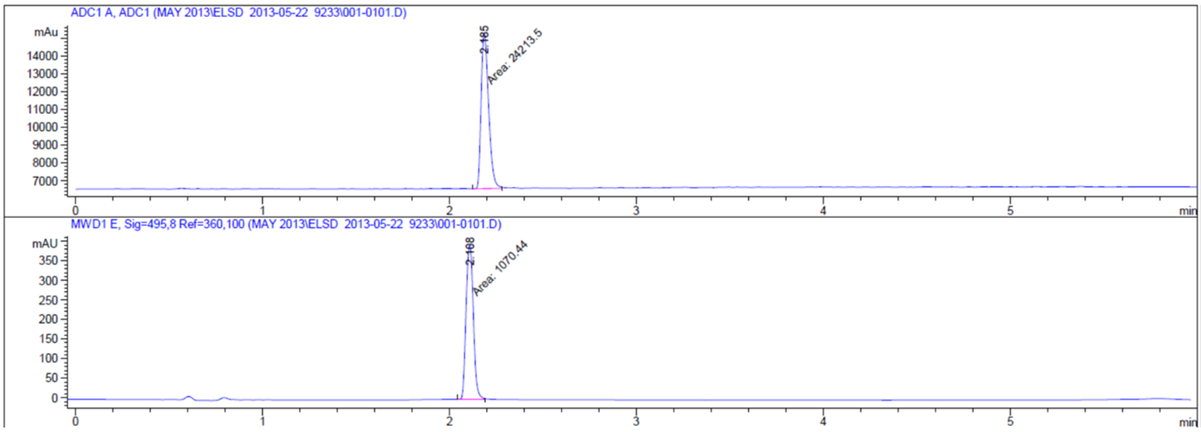
ADC1 A, ADC1 (MAY 2013\ELSD 2013-05-22 9233\001-0101.D)
mAu
2.185
Area: 24213.5
MWD1 E, Sig=495,8 Ref=360,100 (MAY 2013\ELSD 2013-05-22 9233\001-0101.D)
mAU
2.108
Area: 1070.44
min

UBI-7

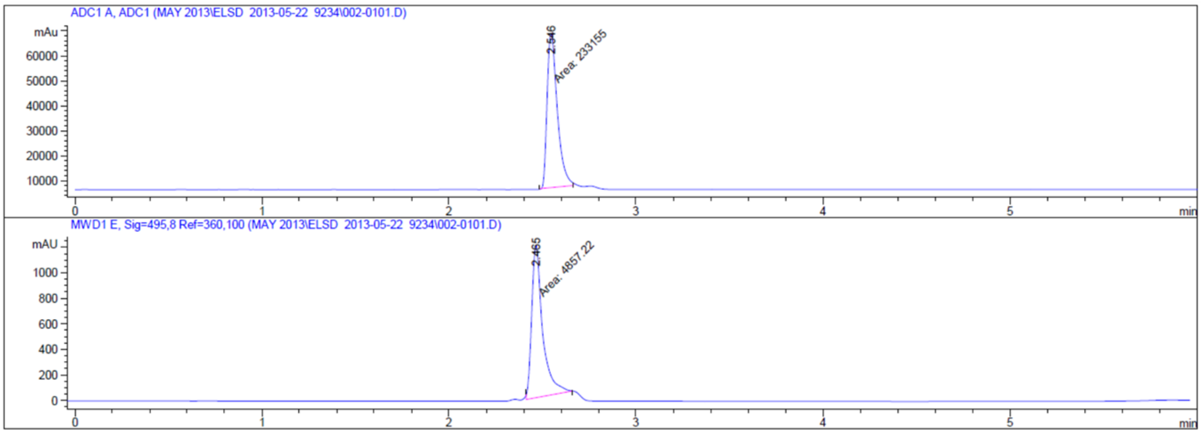
ADC1 A, ADC1 (MAY 2013\ELSD 2013-05-22 9234\002-0101.D)
mAu
2.546
Area: 233155
MWD1 E, Sig=495,8 Ref=360,100 (MAY 2013\ELSD 2013-05-22 9234\002-0101.D)
mAU
2.465
Area: 4857.22
min

UBI-8

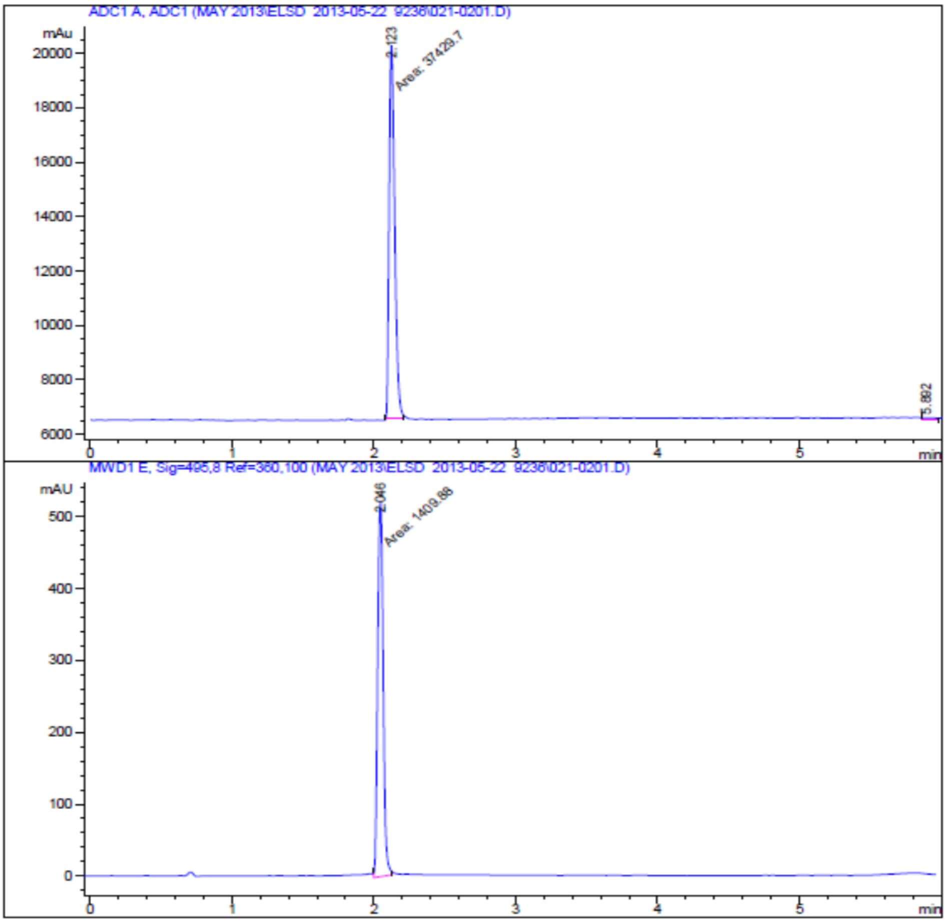
ADC1 A, ADC1 (MAY 2013\ELSD 2013-05-22 9236\021-0201.D)
mAu
2.123
Area: 37429.7
5.892
min
MWD1 E, Sig=495,8 Ref=360,100 (MAY 2013\ELSD 2013-05-22 9236\021-0201.D)
mAU
2.046
Area: 1409.88
min

UBI-9

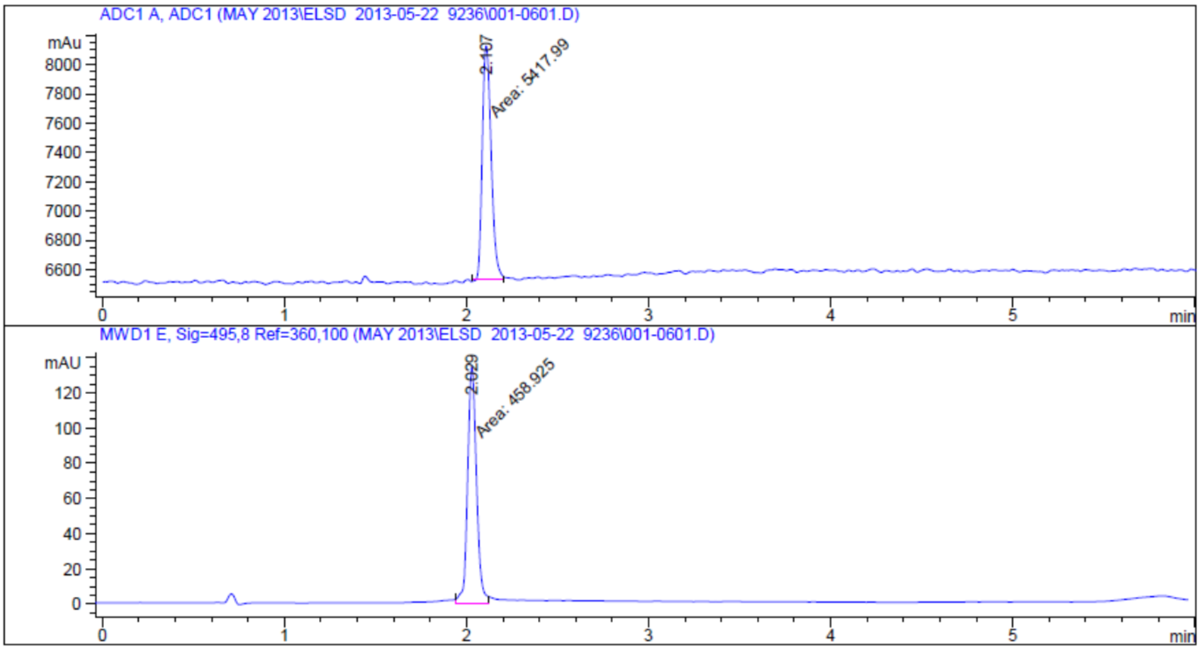
ADC1 A, ADC1 (MAY 2013\ELSD 2013-05-22 9236\001-0601.D)
mAu
2.107
Area: 5417.99
min
MWD1 E, Sig=495,8 Ref=360,100 (MAY 2013\ELSD 2013-05-22 9236\001-0601.D)
mAU
2.029
Area: 458.925
min

UBI-10

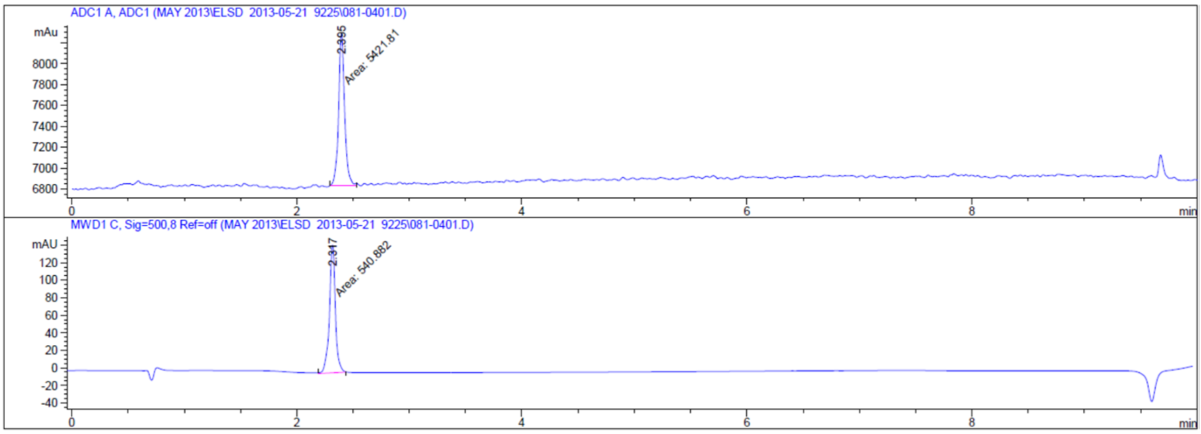

UBI-11

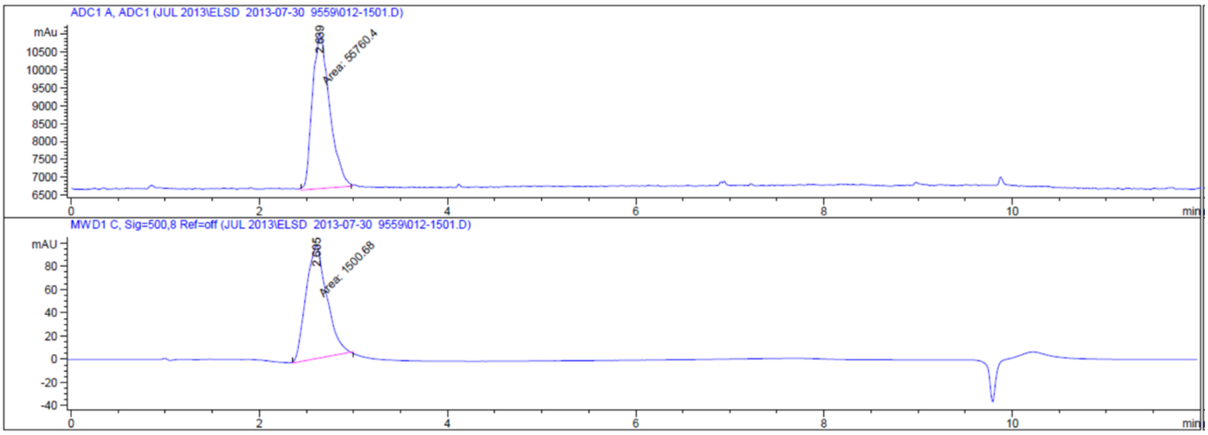

Bacterial Culture and Labelling: Bacteria used were *K. pneumoniae* (ATCC BAA1706) ATCC, *E. coli* (ATCC 25922) ATCC, *A. baumannii* (J3433) Clinical Isolate, *P. aeruginosa* (J3284) Clinical Isolate, Methicillin Resistant *S. aureus* (MRSA) (ATCC25923) ATCC and Methicillin Sensitive *S. aureus* (MSSA) (ATCC 252) ATCC. All bacteria were grown on Lysogeny broth (LB) agar and from a single bacterial colony placed into 10ml of LB and incubated at 37˚C for 16 hours in an orbital shaker. Cultures were centrifuged at 4000rpm for 5 minutes and the pellet resuspended in 1ml phosphate buffered saline (PBS, Life Technologies). Following three further washes in PBS the cultures were reconstituted to 1 OD_{595} nm. Bacteria were counterstained with 5µM Syto 82 orange fluorescent nucleic acid stain (Invitrogen, CA, USA) in a shaking heat block at 37°C for 20 minutes. For FCFM bacteria were stained with 1µM Calcein AM (Sigma, MO, USA) for 30 minutes at 37°C. Counterstained bacteria were washed in PBS to remove excess dye.

Neutrophil Isolation: Neutrophils for *in vitro* experiments were isolated from peripheral venous blood of healthy human volunteers (Ethical approval was obtained from the Lothian Research Ethics Committee). Blood was anticoagulated with sodium citrate (0.38% final concentration) and polymorphonuclear leukocytes were isolated via dextran sedimentation followed by centrifugation through discontinuous plasma-Percoll gradients, as previously described.[S4]

BALF Retrieval: Bronchoalveolar lavage was performed in the Intensive Care Unit (ICU), as previously described.[S5] Pooled samples from three patients were used for stability assays.

Confocal Analysis and Fluorescence Quantification: 8-Well Lab-Tek II Confocal Chambers (VWR, PA, USA) were coated in fibronectin (for neutrophil experiments) or poly-d-lysine (for bacteria) at 37°C for 20 minutes, then washed in PBS prior to bacterial innoculation. For co-culture assays, neutrophils were seeded on fibronectin coated wells at 1 x 10^5 neutrophils per well for 20 minutes and non-adherent neutrophils aspirated. Bacteria were added to each chamber to a final concentration of 0.5 OD_{595} nm with the desired final concentration of Smartprobe. A laser-scanning confocal imaging system (LSM510; Carl Zeiss, Jena, Germany), incorporating an upright Axioskop FS2 microscope (63× objective) was used for image acquisition and processing. Exposure to 488 nm light was limited to 5% of the maximum laser power in order to minimize toxicity. 'Green' fluorescence (for FAM and NBD) was excited with a dedicated 488 nm line and emitted light detected with meta detector (500-530 nM). Syto nuclear and dyes were excited with a dedicated 543 nm line, and emitted light detected with meta detector (570-610 nM). Fields of view were chosen based on the counterstain and at least three fields of view were recorded for each condition. For affinity assays, following initial imaging, the fluid was aspirated and two gentle washes with PBS were performed. The chamber was re-imaged on confocal.

Analysis was with ImageJ (version 1.46r, National Institutes of Health, USA); the Syto channel was automatically thresholded (MaxEntropy, with a set threshold 0-47) and an ROI generated from this. The

mean fluorescence intensity on the probe channel within this ROI was quantified. Data presented represents the mean of three experiments, from each of which three separate fields of view were assessed. All experiments were performed at least three times unless otherwise stated and results expressed as mean ± SEM. Data was analysed by unpaired t-test and significance was determined as $p<0.05$ (GraphPad Prism version 5.01 for Windows, GraphPad Software, San Diego California USA).

***Ex vivo* Human Lung and FCFM Procedure:** Human lung samples were obtained from patients undergoing surgical resection for lung carcinoma. All images were obtained on sections of normal lung away from the cancerous growth. Informed consent was obtained and the study was approved by the Regional Ethics Committee. Samples were taken fresh from the operating theatre, dissected into 4mm sections and placed in wells of 96 well plate. Bacteria were pre-labelled with Smartprobe or Calcein AM and co-cultured in the well with a maximum volume of 100µl. Wells were imaged with a clinically approved FCFM system using a confocal Alveoflex miniprobe and 488nm laser scanning unit (Cellvizio, Mauna Kea Technologies, Paris). Images were obtained using 100% laser power, frame rate of 12 frames per second and image intensity thresholding was equivalent across experiments.

References

S1. E. Kaiser, R. L. Colescott, C. D. Bossinger and P. I. Cook, *Anal. Biochem.* **1970**, 34, 595-598.

S2. T. Vojkovsky, *Peptide research*, 1995, **8**, 236-237.

S3. R. Fischer, O. Mader, G. Jung and R. Brock, *Bioconjugate chemistry*, 2003, **14**, 653-660.

S4. C. Haslett, L. A. Guthrie, M. M. Kopaniak, R. B. Johnston, Jr. and P. M. Henson, *The American journal of pathology*, 1985, **119**, 101-110; A. G. Rossi, J. C. McCutcheon, N. Roy, E. R. Chilvers, C. Haslett and I. Dransfield, *J Immunol*, 1998, **160**, 3562-3568.

S5. A. Conway Morris, K. Kefala, T. S. Wilkinson, O. L. Moncayo-Nieto, K. Dhaliwal, L. Farrell, T. S. Walsh, S. J. Mackenzie, D. G. Swann, P. J. Andrews, N. Anderson, J. R. Govan, I. F. Laurenson, H. Reid, D. J. Davidson, C. Haslett, J. M. Sallenave and A. J. Simpson, *Thorax*, 2010, **65**, 201-207.
